# Supplementary material for: CRISPR-Cas12a induced DNA double-strand breaks are repaired by multiple pathways with different mutation profiles in Magnaporthe oryzae
Source: Nat Commun. 2022 Nov 22;13:7168. doi: 10.1038/s41467-022-34736-1 (PMC9684475; doi:10.1038/s41467-022-34736-1)
Supplement: Supplementary file 1 — Supplementary Information [file 41467_2022_34736_MOESM1_ESM.pdf]

CRISPR-Cas12a induced DNA double-strand breaks are repaired by multiple pathways with different mutation profiles in *Magnaporthe oryzae*

Supplementary Information

Jun Huang, David Rowe, Pratima Subedi, Wei Zhang, Tyler Suelter, Barbara Valent,

David E. Cook*

Department of Plant Pathology, Kansas State University, Manhattan, KS 66506-5502, USA

* Correspondence to: decook@ksu.edu

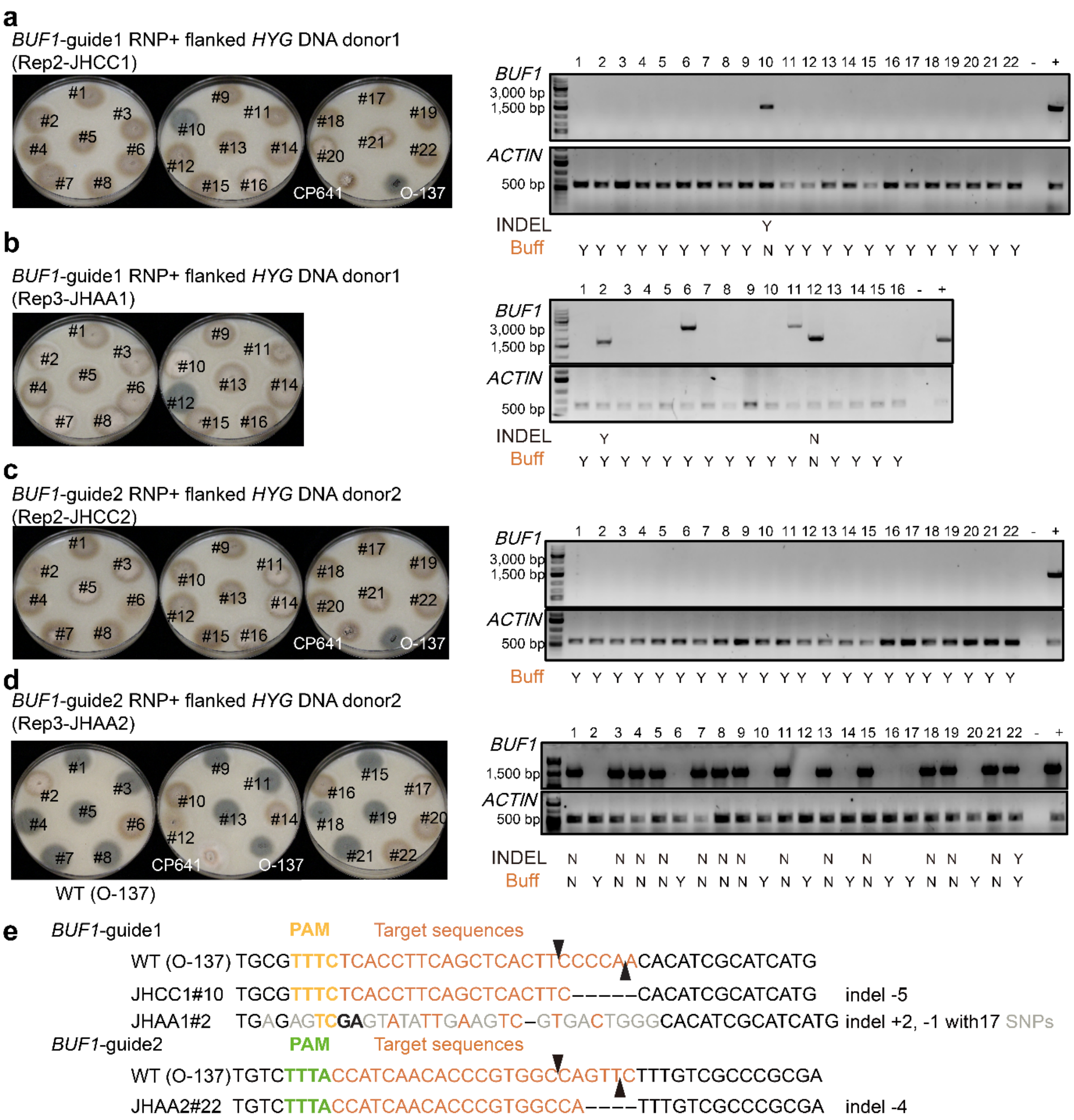

Supplementary Fig. 1 Cas12a RNP combined with short homology donor DNA cause efficient gene deletion at *BUF1* locus in the field isolate O-137.

(a, b, c, and d) After protoplast transformations, hygromycin resistant transformants from replications 2 and 3 were plated in OTA for phenotyping and genotyping. DNA of transformants was extracted for genotyping with *BUF1*_F/R primer pair. *ACTIN* amplification served as the loading control. CP641 derived from O-137, is the control strain for showing the buff phenotype, O-137 is the wild-type isolate used in these assays. - and + indicate the negative control (water) and positive control (O-137 genomic DNA) for PCR amplification, respectively. (e) The detailed sequences change of the mutants with INDELs are presented. Yellow (*BUF1*-guide1)/green (*BUF1*-guide2) and orange letters highlight PAM and target sequences,

respectively. Black triangles around the target sequences highlight the predicted Cas12a cut site. Grey letters indicate the observed SNPs.

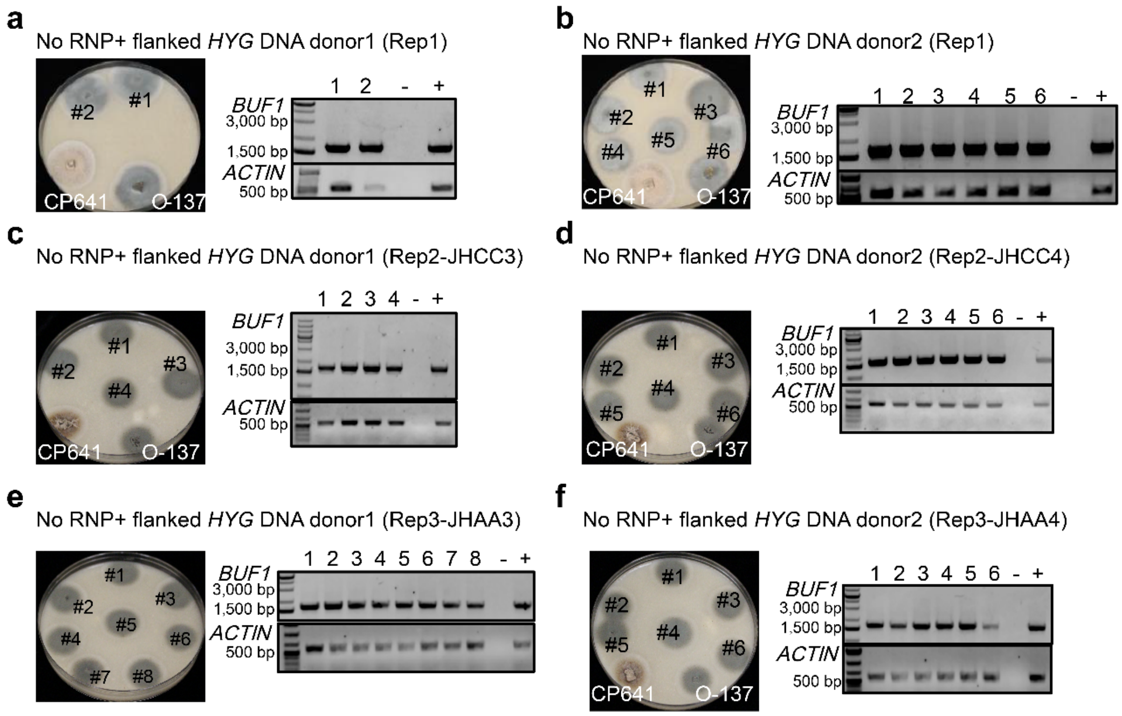

Supplementary Fig. 2 Flanked *HYG* DNA donor alone cannot cause the buff mutation.
(a, c and e) Hygromycin resistant transformants from three biological replications generated with flanked *HYG* DNA donor1 alone were plated on OTA for phenotyping. CP641 derived from O-137, is the control strain for showing the buff phenotype, O-137 is the wild-type isolate used in these assays. Genotyping result with *BUF1* primers shows the indistinguishable size of amplifications with wild-type (+). Water (-) was used as the negative control. *ACTIN* amplification served as the loading control. Similar assays performed with flanked *HYG* DNA donor2 are showed in panel (b, d and f).

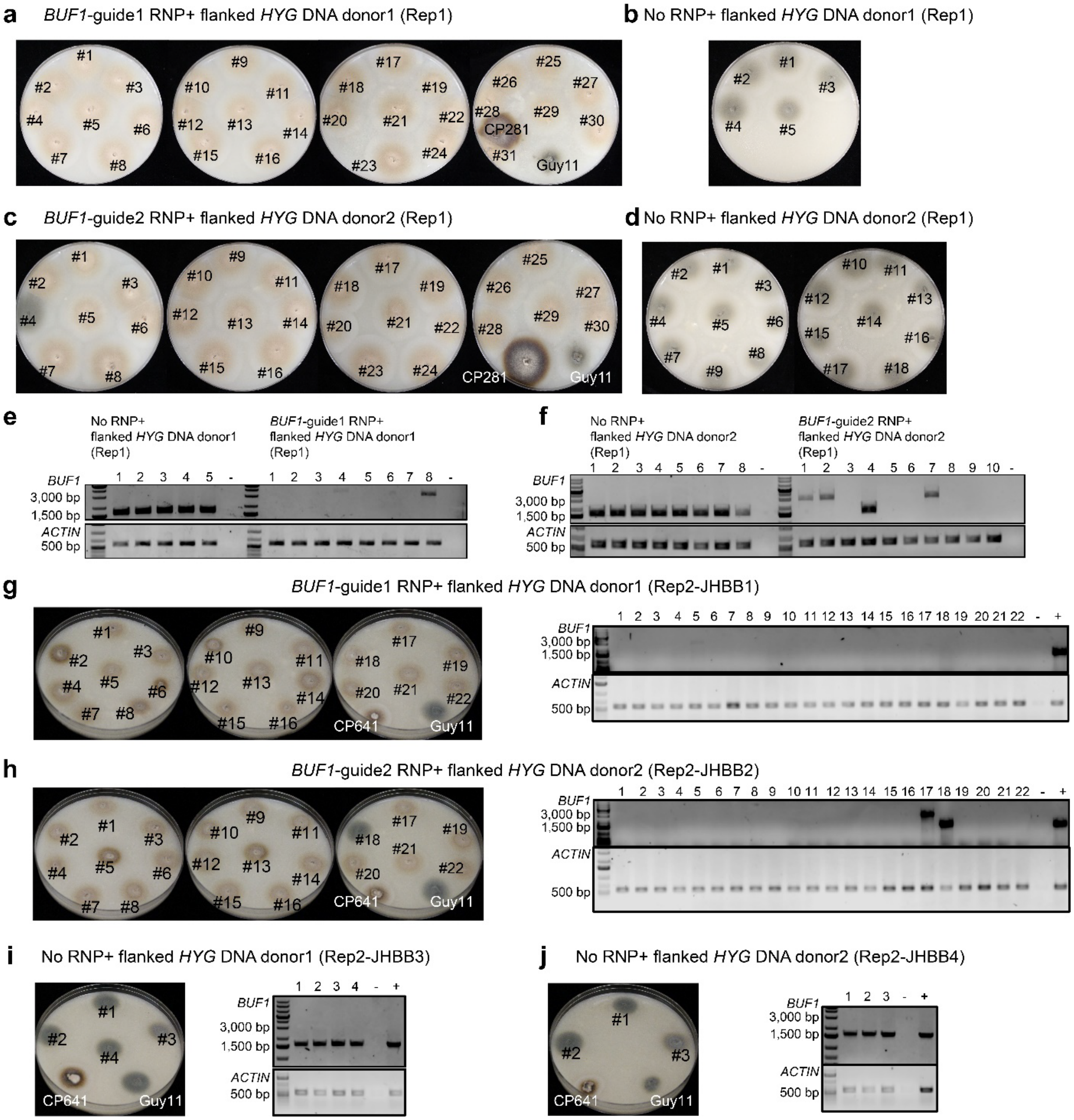

Supplementary Fig. 3 Cas12a RNP combined with short homology donor DNA cause efficient gene deletion at *BUF1* locus in the field isolate Guy11.

(a, c, g, h, i and j) After protoplast transformations, hygromycin resistant transformants from biological replications were plated in RPA or OTA for phenotyping. (e, f, g, h, i and j) DNA of selected transformants were extracted for genotyping with *BUF1*_F/R primer pair. *ACTIN* amplification served as the loading control. CP281 (a weeping lovegrass strain) or CP641 (derived from O-137) showing the buff phenotype, Guy11 is the wild-type isolate used in these assays. - and + indicate the negative control (water) and positive control (Guy11 genomic DNA) for PCR amplification, respectively. Two independent transformations with further genotyping were performed for each assay or control.

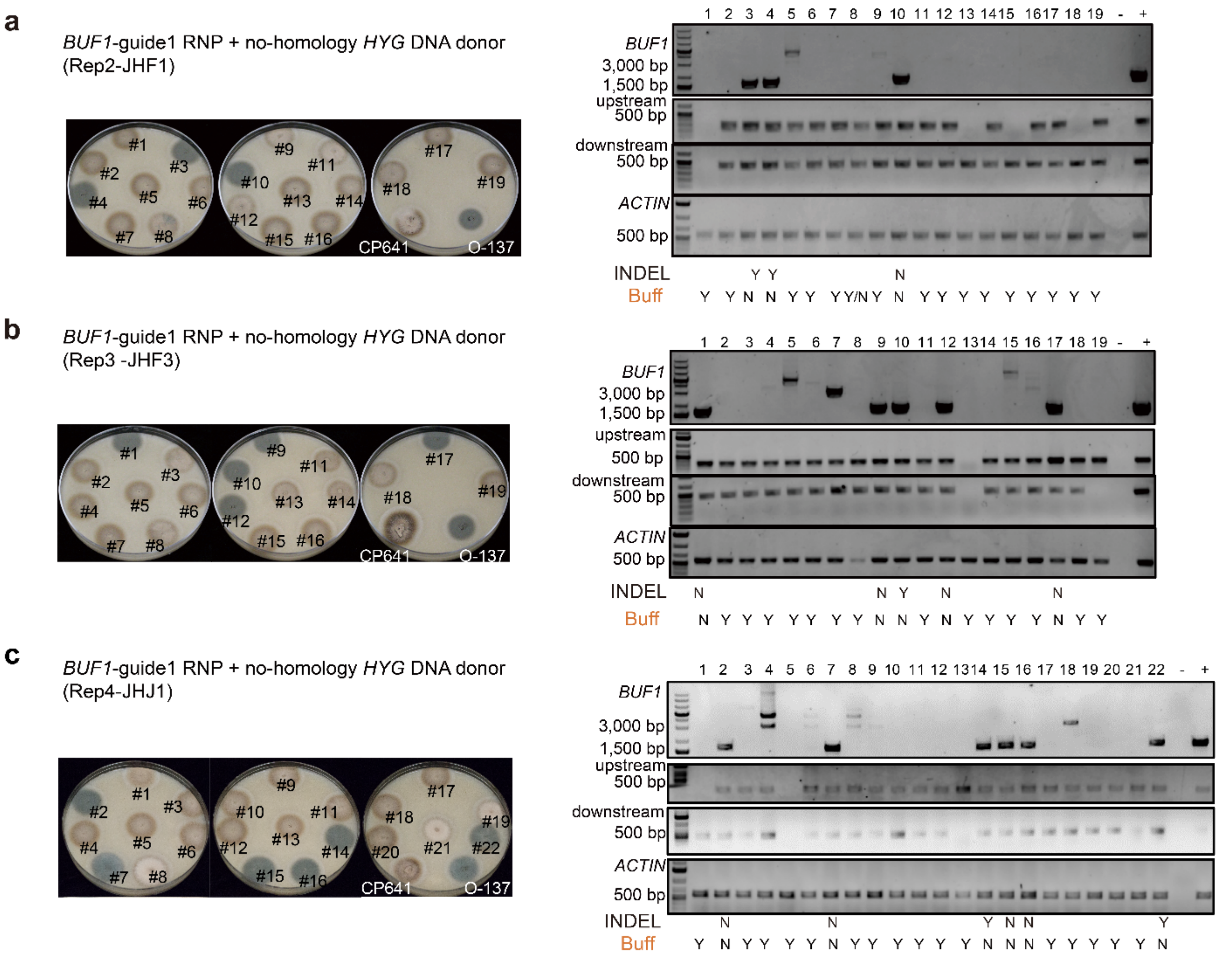

Supplementary Fig. 4 No-homology donor DNA alone with *BUF1*-guide1 RNP causes buff mutation.

(a, b and c) Hygromycin resistant transformants with *BUF1*-guide1 RNP and no-homology *HYG* DNA donor generated from three biological assays (rep 2 to 4) were placed on OTA for testing the mycelial color change. CP641 is the control for buff color change, O-137 is the wild-type isolate used in the experiment. Genotyping results are shown in the right panel. The wild-type-like PCR products were purified and Sanger sequenced to detect the potential INDELs. INDEL N indicates there were no INDELs observed after sequencing, otherwise labelled as INDEL Y. Buff Y indicates the strains used for genotyping were buff, while Buff N indicates wild-type phenotype. - and + indicate the negative control (water) and positive control (O-137 genomic DNA) for PCR amplification, respectively. Upstream and downstream primer pairs were used for detecting the existence of large-scale locus alternation. *ACTIN* amplification served as the loading control.

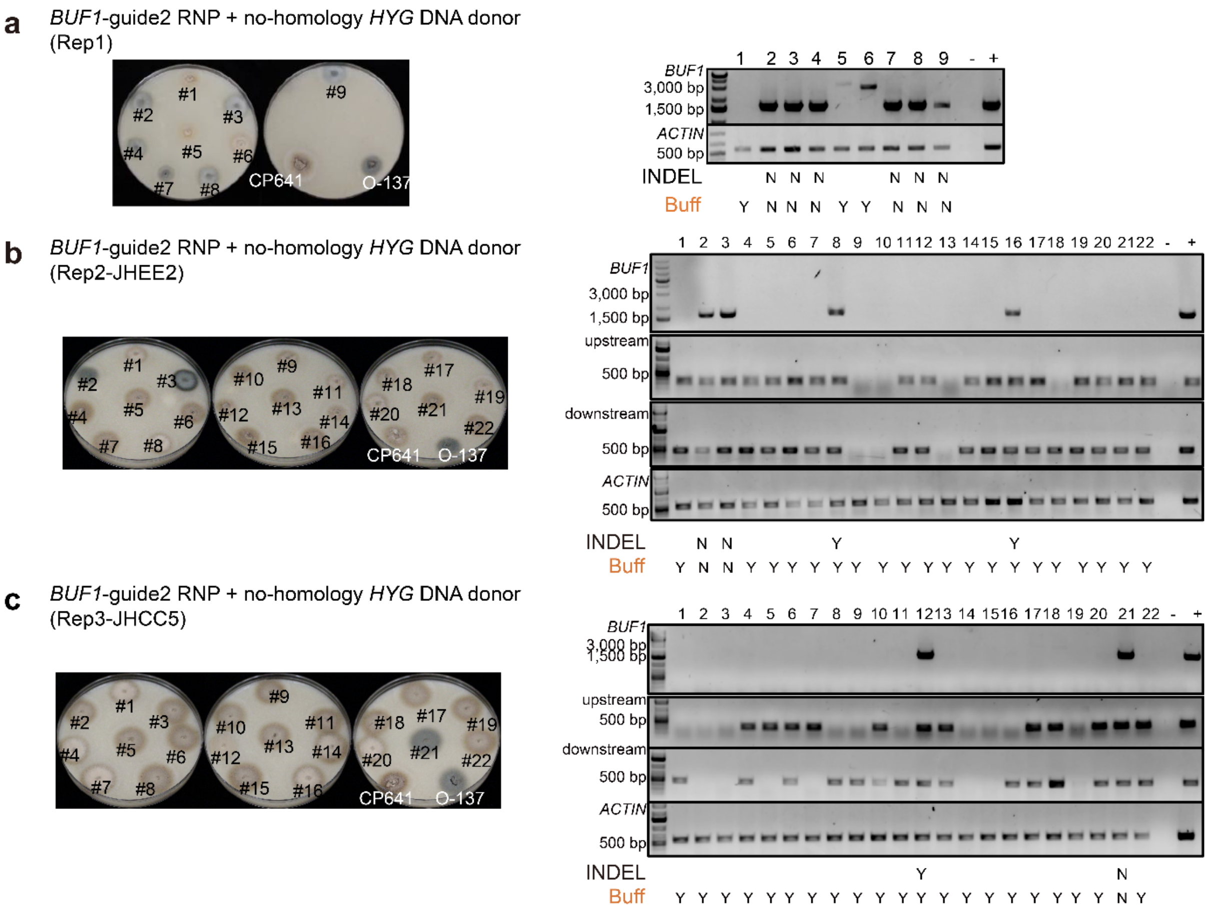

Supplementary Fig. 5 No-homology donor DNA alone with *BUF1*-guide2 RNP causes buff mutation.

(a, b and c) Hygromycin resistant transformants with *BUF1*-guide2 RNP and no-homology *HYG* DNA donor generated from three biological assays (rep 1 to 3) were placed on OTA for testing the mycelial color change. CP641 is the control for buff color change, O-137 is the wild-type isolate used in the experiment. Genotyping results are shown in the right panel. The wild-type-like PCR products were purified and Sanger sequenced to detect the potential INDELs. INDEL N indicates there were no INDELs observed after sequencing, otherwise labelled as INDEL Y. Buff Y indicates the strains used for genotyping were buff, while Buff N indicates wild-type phenotype. - and + indicate the negative control (water) and positive control (O-137 genomic DNA) for PCR amplification, respectively. Upstream and downstream primer pairs were used for detecting the existence of large-scale locus alternation. *ACTIN* amplification served as the loading control.

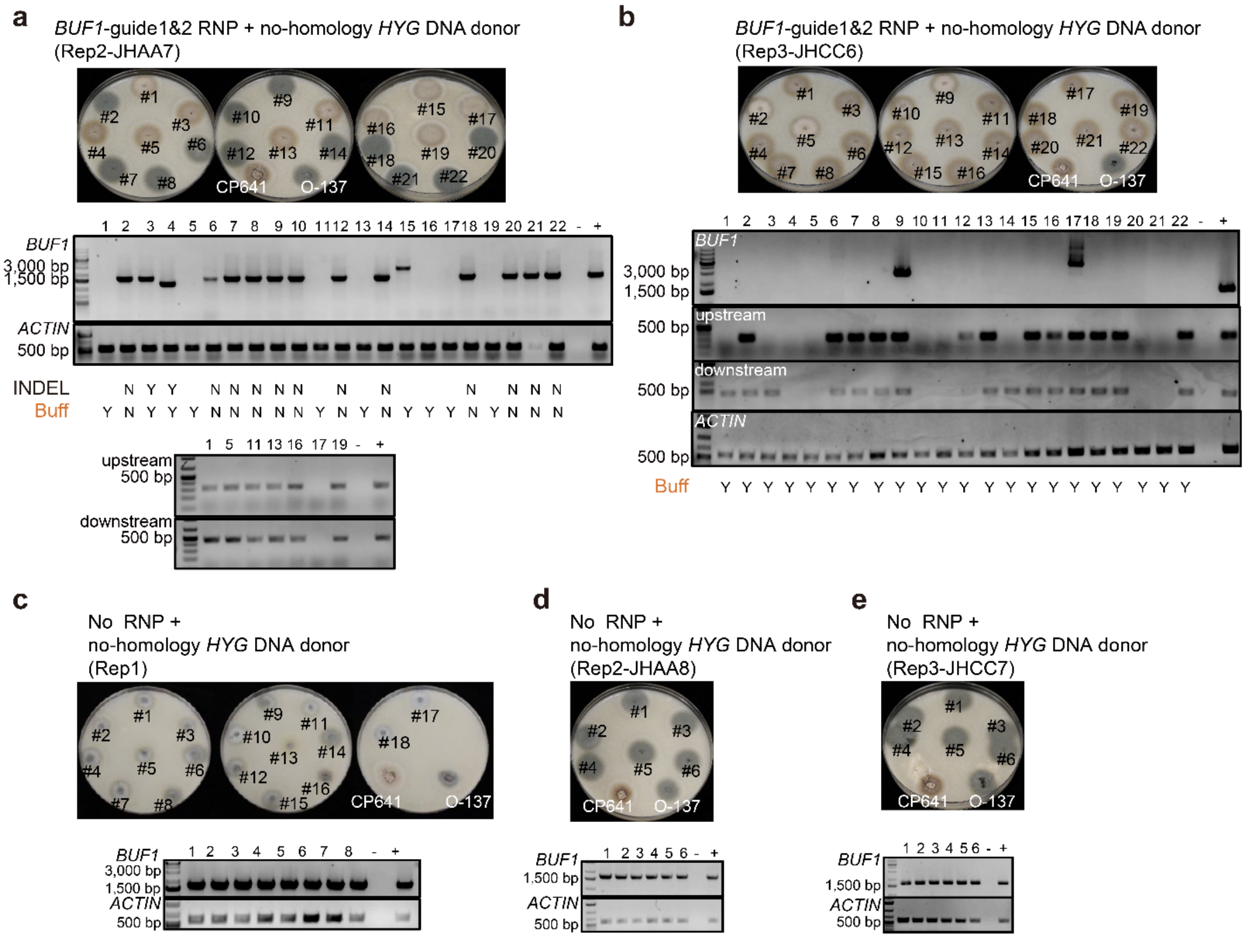

Supplementary Fig. 6 No-homology donor DNA alone with dual *BUF1*- RNP causes buff mutation, while DNA donor alone cannot cause the buff mutation.

(a and b) Hygromycin resistant transformants with *BUF1*-guide1&2 RNP and no-homology *HYG* DNA donor generated from two biological assays (rep 2 and 3) were placed on OTA for testing the mycelial color change. CP641 is the control for buff color change, O-137 is the wild-type isolate used in the experiment. Genotyping results are shown in the bottom panel. The wild-type-like PCR products were purified and Sanger sequenced to detect the potential INDELs. INDEL N indicates there were no INDELs observed after sequencing, otherwise labelled as INDEL Y. Buff Y indicates the strains used for genotyping were buff, while Buff N indicates wild-type phenotype. - and + indicate the negative control (water) and positive control (O-137 genomic DNA) for PCR amplification, respectively. Upstream and downstream primer pairs were used for detecting the existence of large-scale locus alternation. *ACTIN* amplification served as the loading control. Similar assays performed with no-homology *HYG* DNA alone are showed in panel (c, d and e).

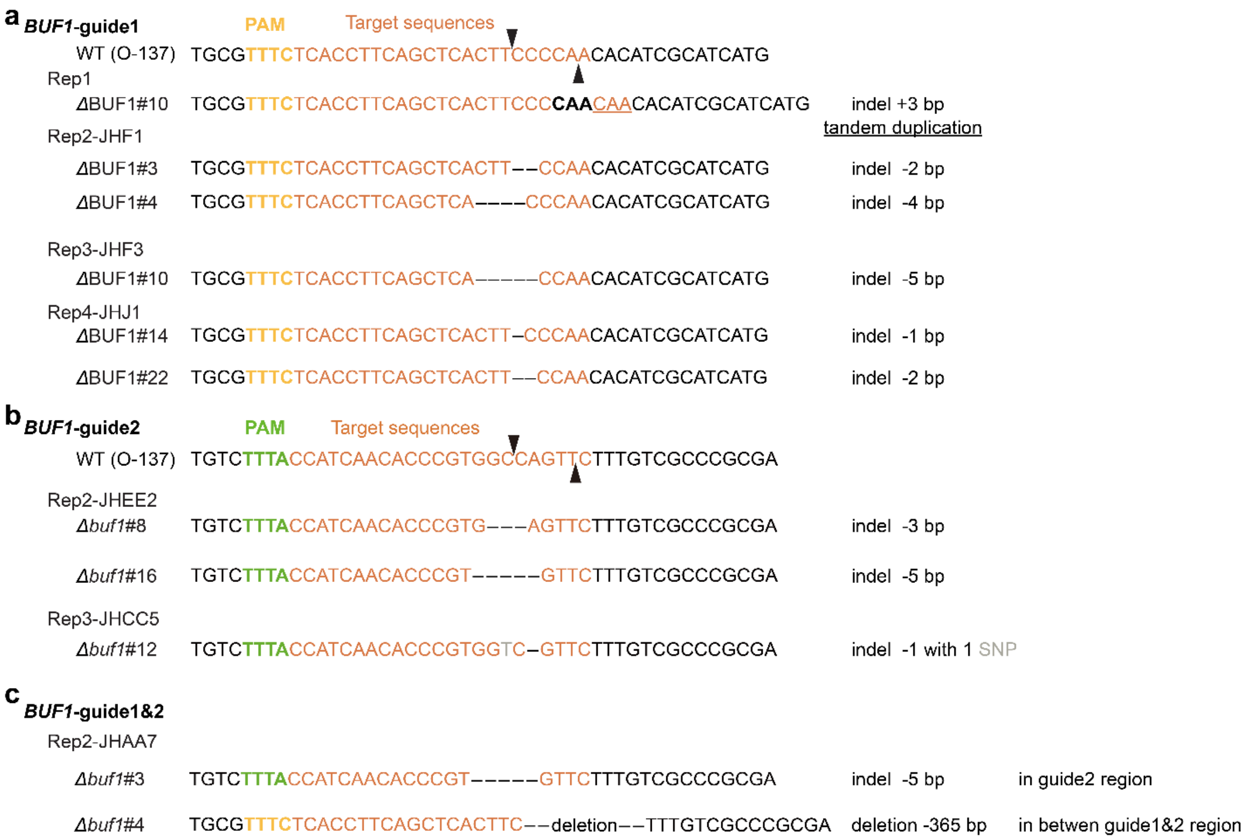

Supplementary Fig. 7 INDELs are detected in edited strains from O-137.
Yellow (guide1)/green (guide2) and orange letters highlight PAM and target sequences, respectively. Black triangles around the target sequences highlight the potential Cas12a cut site. Underlined letters indicate the potential template for tandem duplication. Grey letters indicate the observed SNPs. The INDEL mutations found with *BUF1*-guide1, *BUF1*-guide2 and dual targeting are presented in (a), (b) and (c), respectively.

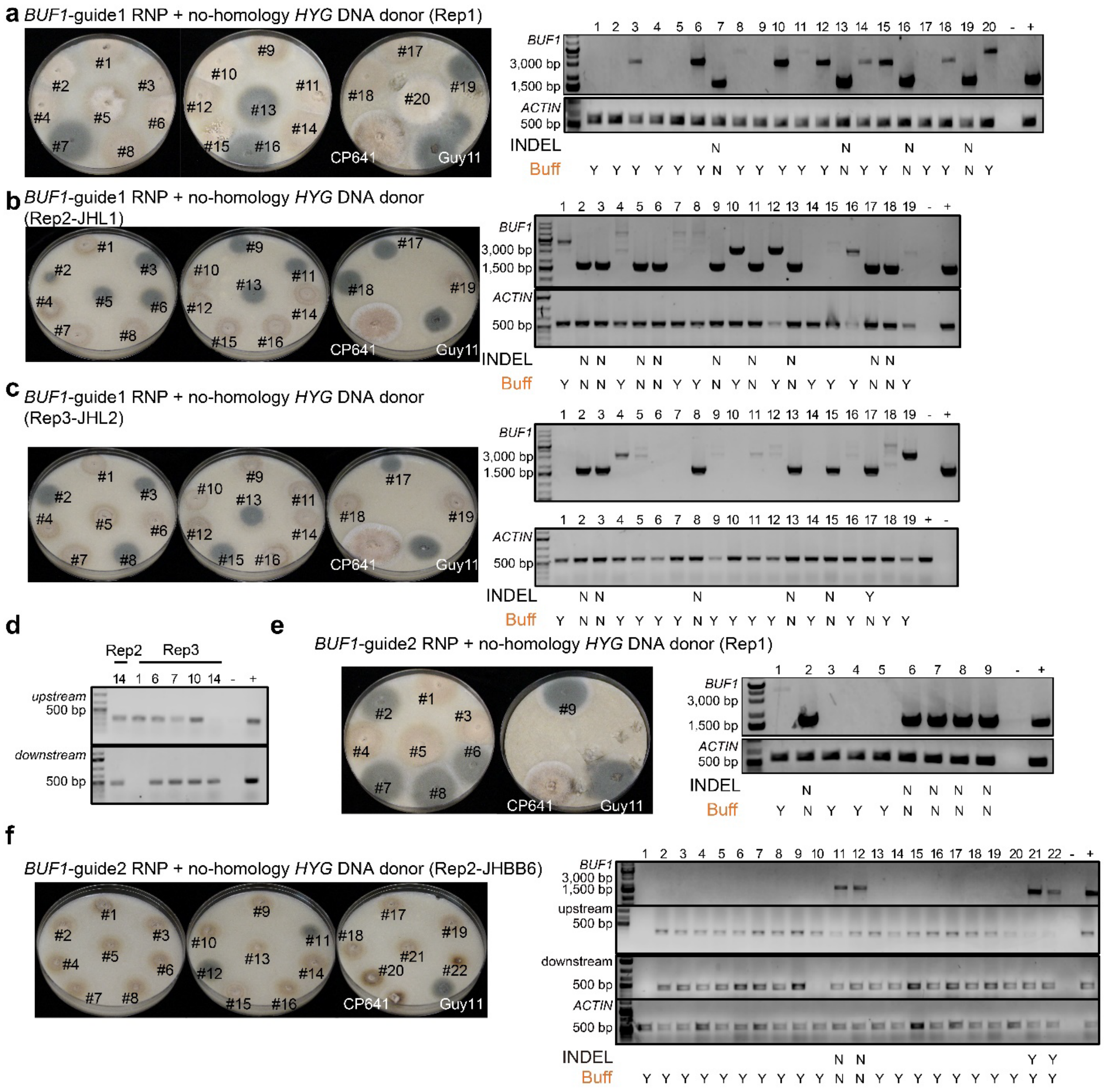

Supplementary Fig. 8 Donor DNA without homology sequences integrates at Cas12a target site in the genetic background of Guy11.

(a, b and c) Hygromycin resistant transformants with *BUF1*-guide1 RNP and no-homology *HYG* DNA donor from three biological assays (rep 1 to 3) were plated on OTA for phenotyping. CP641 is a control for buff color change, Guy11 is the wild-type isolate used in the experiment. *BUF1*_F/R primer pair was used for genotyping. *ACTIN* amplification was served as the loading control. - and + indicate the negative control (water) and positive control (Guy11 genomic DNA) for PCR amplification, respectively. The wild-type-like PCR products were purified and Sanger sequenced to detect the INDELs. INDEL N indicates there were no INDELs observed after sequencing, otherwise labelled as INDEL Y. Buff Y indicates the strains used for genotyping were buff, while Buff N indicates wild-type phenotype. (d) Upstream and downstream primer pairs were used for detecting the existence of large-scale locus alternation from two independent

transformations. Similar layouts for different transformants are presented in (e and f) transformants with *BUF1*-guide2 RNP and no-homology *HYG* DNA donor from two independent biological replications (rep1 and 2).

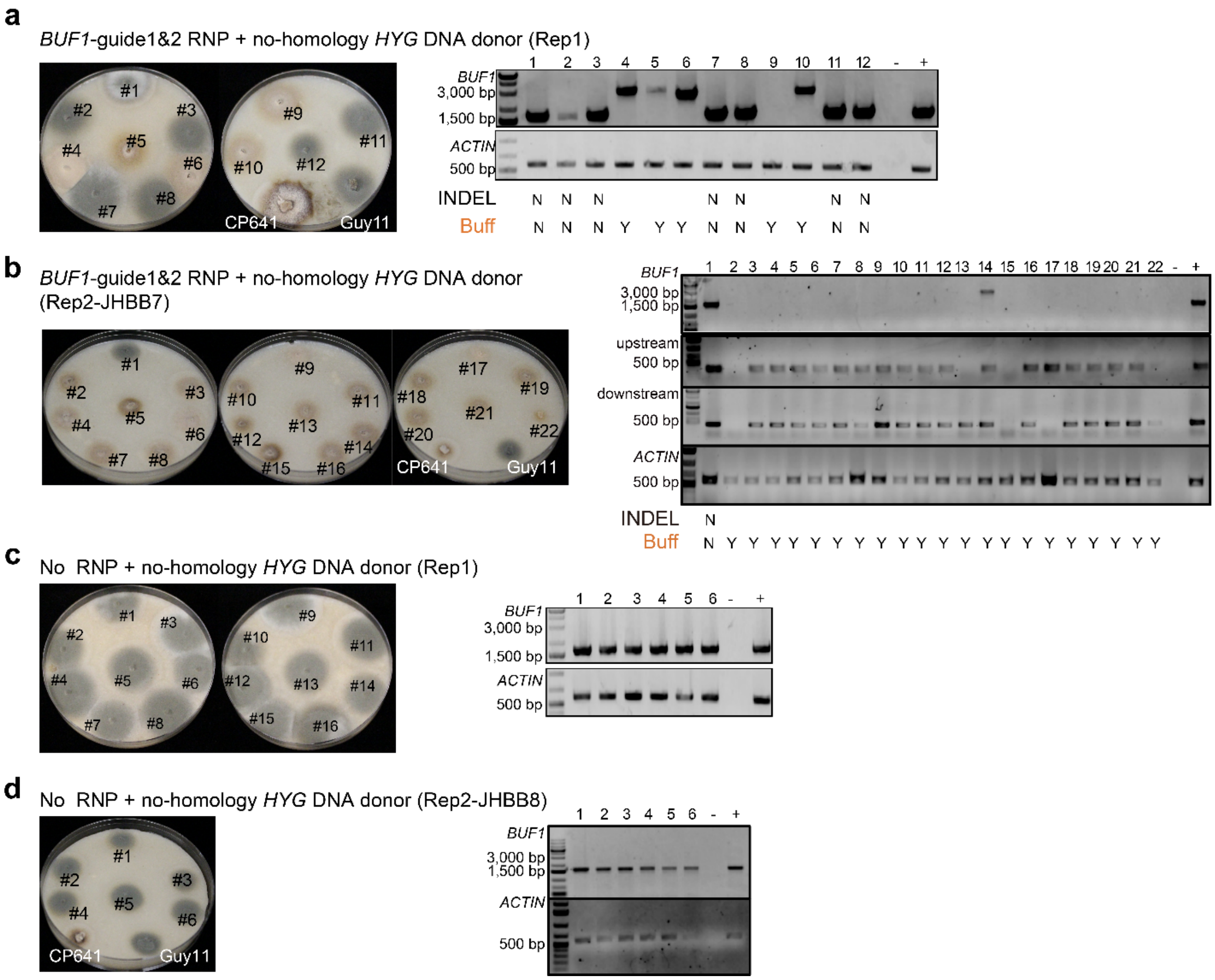

Supplementary Fig. 9 No-homology donor DNA alone with dual *BUF1*- RNP causes buff mutation, while DNA donor alone cannot cause the buff mutation in the genetic background of Guy11.

(a and b) Hygromycin resistant transformants with dual *BUF1*-guide1/2 RNPs and no-homology *HYG* DNA donor generated from two biological assays (rep 1 and 2) were placed on OTA for testing the mycelial color change. CP641 is the positive control for buff color change, Guy11 is the wild-type isolate used in the experiment. Genotyping results are shown in the right panel. The wild-type-like PCR products were purified and Sanger sequenced to detect the potential INDELs. INDEL N indicates there were no INDELs observed after sequencing, otherwise labelled as INDEL Y. Buff Y indicates the strains used for genotyping were buff, while Buff N indicates wild-type phenotype. - and + indicate the negative control (water) and positive control (Guy11 genomic DNA) for PCR amplification, respectively. Upstream and downstream primer pairs were used for detecting the existence of large-scale locus alternation. *ACTIN* amplification served as the loading control. Similar assays performed with no-homology *HYG* DNA alone are showed in panel (c and d).

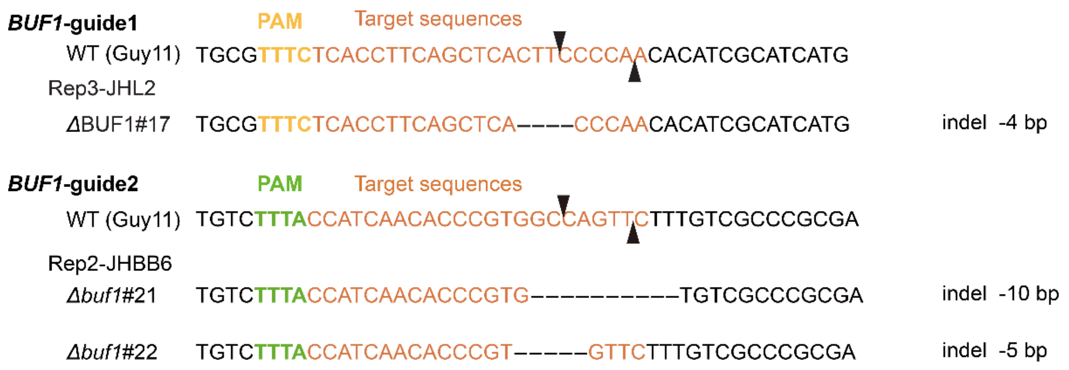

Supplementary Fig. 10 INDELs are detected in edited strains from Guy11.
Yellow (guide1), green (guide2) and orange letters highlight PAM and target sequences, respectively. Black triangles around the target sequences highlight the predicted Cas12a cut site.

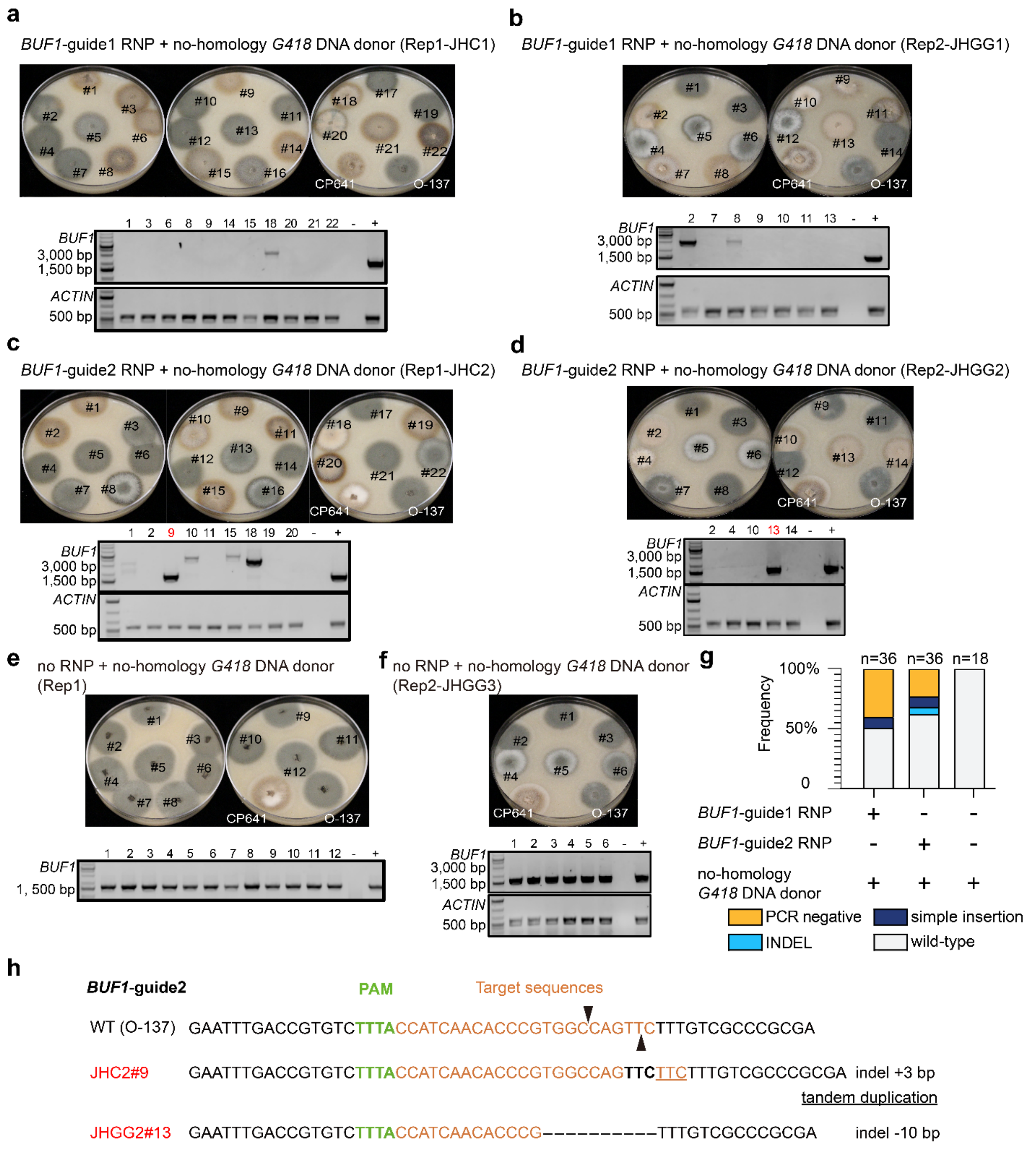

Supplementary Fig. 11 Non-canonical DNA repair at *BUF1* locus is donor and antibiotic independent.

(a and b) G418 resistant transformants with *BUF1*-guide1 RNP and no-homology *G418* DNA donor from two independent biological replications (rep1 and 2) were plated on OTA for phenotyping. Buff mutants were used for DNA extraction and further genotyping with *BUF1*_F/R primer pair. *ACTIN* amplification served as the loading control. Same assays but with *BUF1*-guide2 RNP and no-homology *G418* DNA donor or donor alone are presented in (c and d) and (e and f), respectively. CP641 is a control for buff color change, O-137 is the wild-type isolate used in the

experiment. - and + indicate the negative control (water) and positive control (O-137 genomic DNA) for PCR amplification, respectively. (g) The frequency summary of DNA DSB repair outcomes in O-137. The number of independent PCR validated transformants (x) is listed (n=x). The mutation outcomes were concluded from two biological replications. The frequencies show the average outcome across replicates. Raw data counts are provided in the Source Data file. (h) DNA from JHC2#9 from (c) and JHGG2#13 from (d) as highlighted by red were subjected to Sanger sequencing and aligned with wild-type sequence. Underlined letters indicate the potential template for tandem duplication.

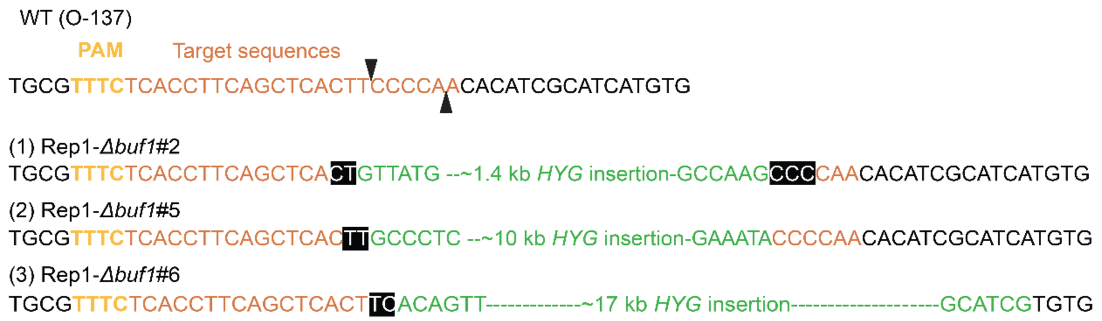

Supplementary Fig. 12 ***HYG*** **DNA donor shares microhomology with original** ***BUF1*** **DNA sequences in the sequenced simple and large insertion mutants.**

2-3 bp microhomology shared between *HYG* DNA donor and original *BUF1* DNA sequences are highlighted by white letters with black boxes.

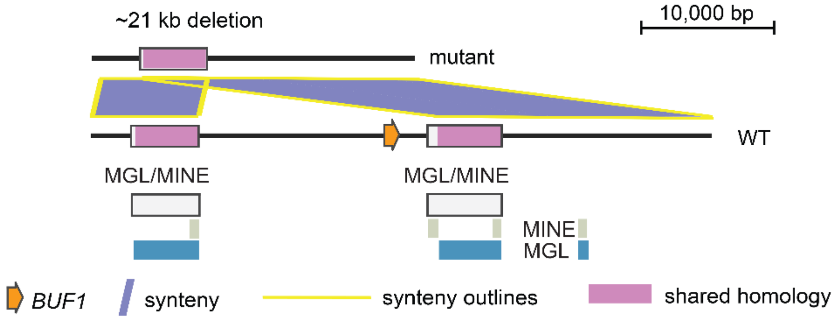

Supplementary Fig. 13 Composition of MGL/MINE in the large deletion mutant.
Presumably the sequence similarity between two hybrid MGL/MINE was used to resolve the break resulting in a single retrotransposon copy and the 21 kb deletion.

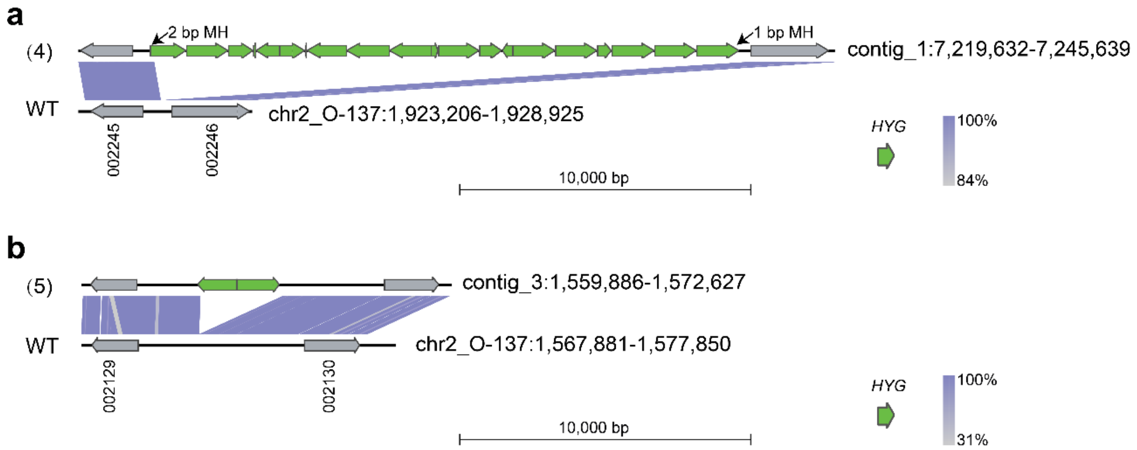

Supplementary Fig. 14 Insertion site of no-homology *HYG* DNA donor in the large deletion mutants.

(a) (b) Synteny illustration of insertion site of no-homology *HYG* DNA donor in the large deletion mutants ((4) Rep1-*Δbuf1#*4 and (5) Rep4-*Δbuf1#*5 from Fig. 3c) are presented. Note: there is a short contig (contig_73, (20,048bp)) in Rep4-*Δbuf1#*5 showing the same *HYG* DNA donor insertion site, but with 5 copies of no-homology *HYG* DNA donor inserted.

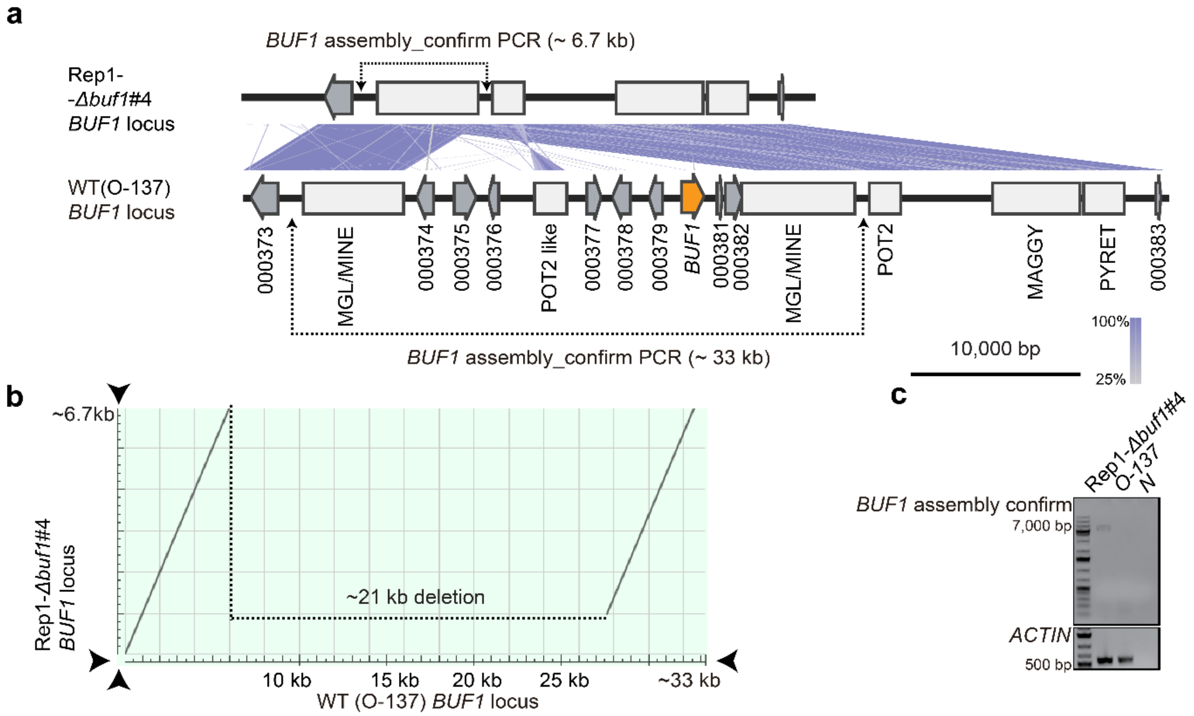

Supplementary Fig. 15 Confirmation of accurate assembly for a large deletion mutant by PCR amplification.
(a) Illustration of *BUF1* locus in wild-type (O-137) and Rep1-*Δbuf1#4* ((4) from Fig. 3c). *BUF1* assembly_confirm PCR primers were used to test the accuracy of assembly. (b) The BLAST result of *BUF1* locus in two above-described strains (query: *BUF1* locus in Rep1-*Δbuf1#4,* (4) from Fig. 3c, subject: *BUF1* locus in wild-type (O-137), the black arrow indicates where the *BUF1* assembly_confirm PCR primers locate. (c) PCR amplification confirms the large deletion in Rep1-*Δbuf1#4*, N indicates the negative control (water). The assay was repeated twice independently with similar results.

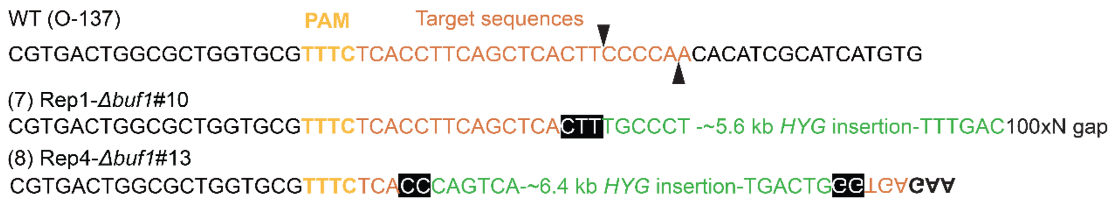

Supplementary Fig. 16 *HYG* DNA donor shares microhomology with original *BUF1* DNA sequences in the deletion plus insertion mutants.

2-3 bp microhomology shared between *HYG* DNA donor and original *BUF1* DNA sequences are highlighted by white letters with black boxes. Deletion plus insertion mutants ((7) Rep1-*Δbuf1#*10 and (8) Rep4-*Δbuf1#*13 from Fig. 3d) are presented.

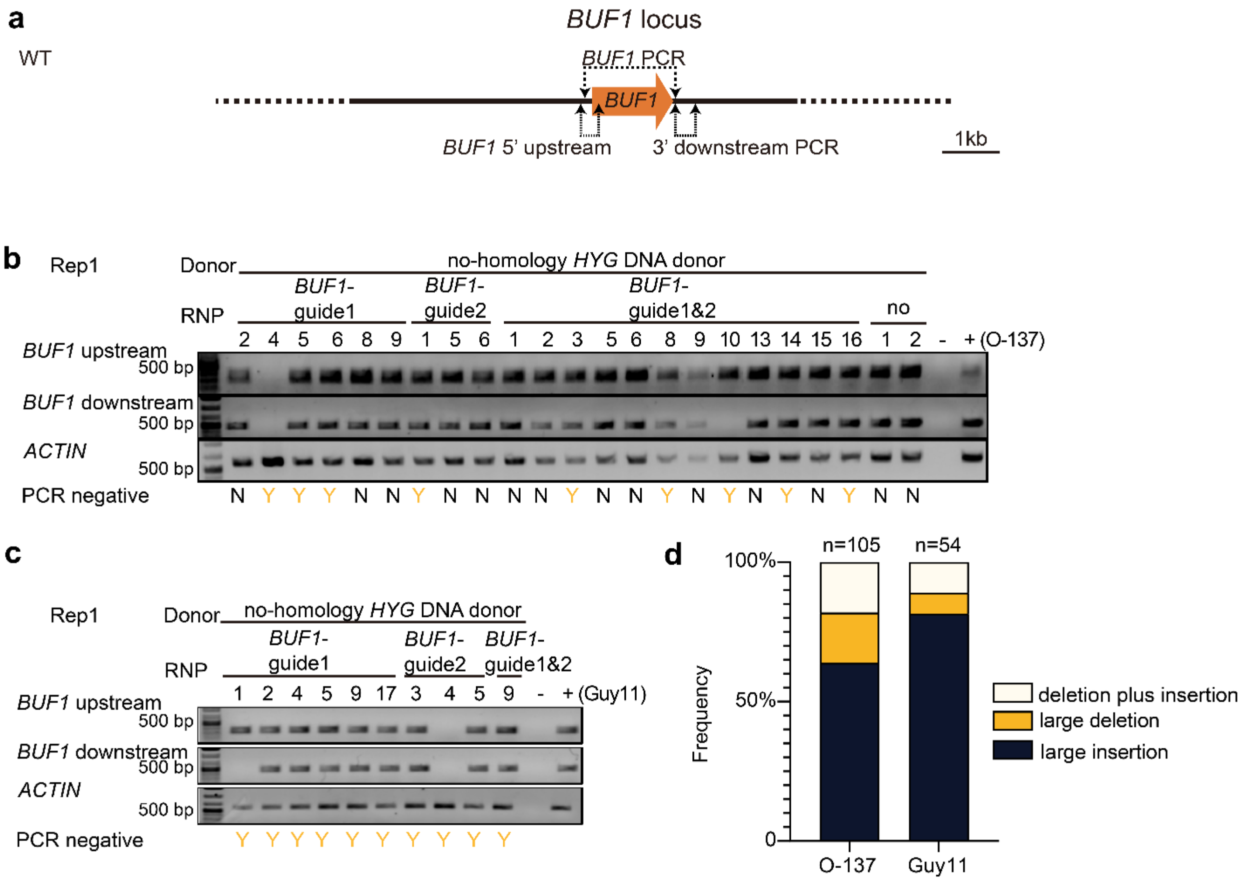

Supplementary Fig. 17 PCR genotyping confirms three non-canonical error-prone DNA repair patterns.

(a) Illustration of *BUF1* locus in the wild-type. (b, c) 5' upstream and 3' downstream regions of *BUF1*, as highlighted in (a) were amplified for testing the occurrence of large insertions, large deletions, or deletion plus insertions at the *BUF1* locus. Black line above the lanes indicates the DNA donor and RNP used for generating the strains. PCR negative Y indicates that PCR negative genotyping result was found in the strain with *BUF1* PCR, otherwise, labelled as PCR negative N. *ACTIN* amplification served as the loading control. – and + indicate the negative control (water) and positive control (O-137 or Guy11 genomic DNA) for PCR amplification, respectively. The assay was repeated twice independently with similar results. (d) The frequency for different non-canonical DNA repair outcomes in O-137 or Guy11 with no-homology *HYG* DNA donor and *BUF1* RNPs. The O-137 data were calculated from at least three biological replications, while the Guy11 data were from at least two biological replications. The number (x) of independent transformants used for counting is listed 'n=x'. The class frequencies are reported as the total across all experiments. The raw data is provided in the Source Data file.

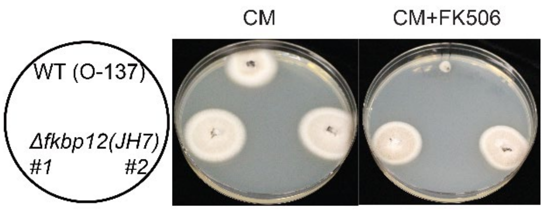

Supplementary Fig. 18 *Δfkbp12* is insensitive to FK506.
Representative phenotyping result of *Δfkbp12*. CM supplemented with 1 µg/mL FK506 was used for testing the sensitivity of distinct strains to FK506.

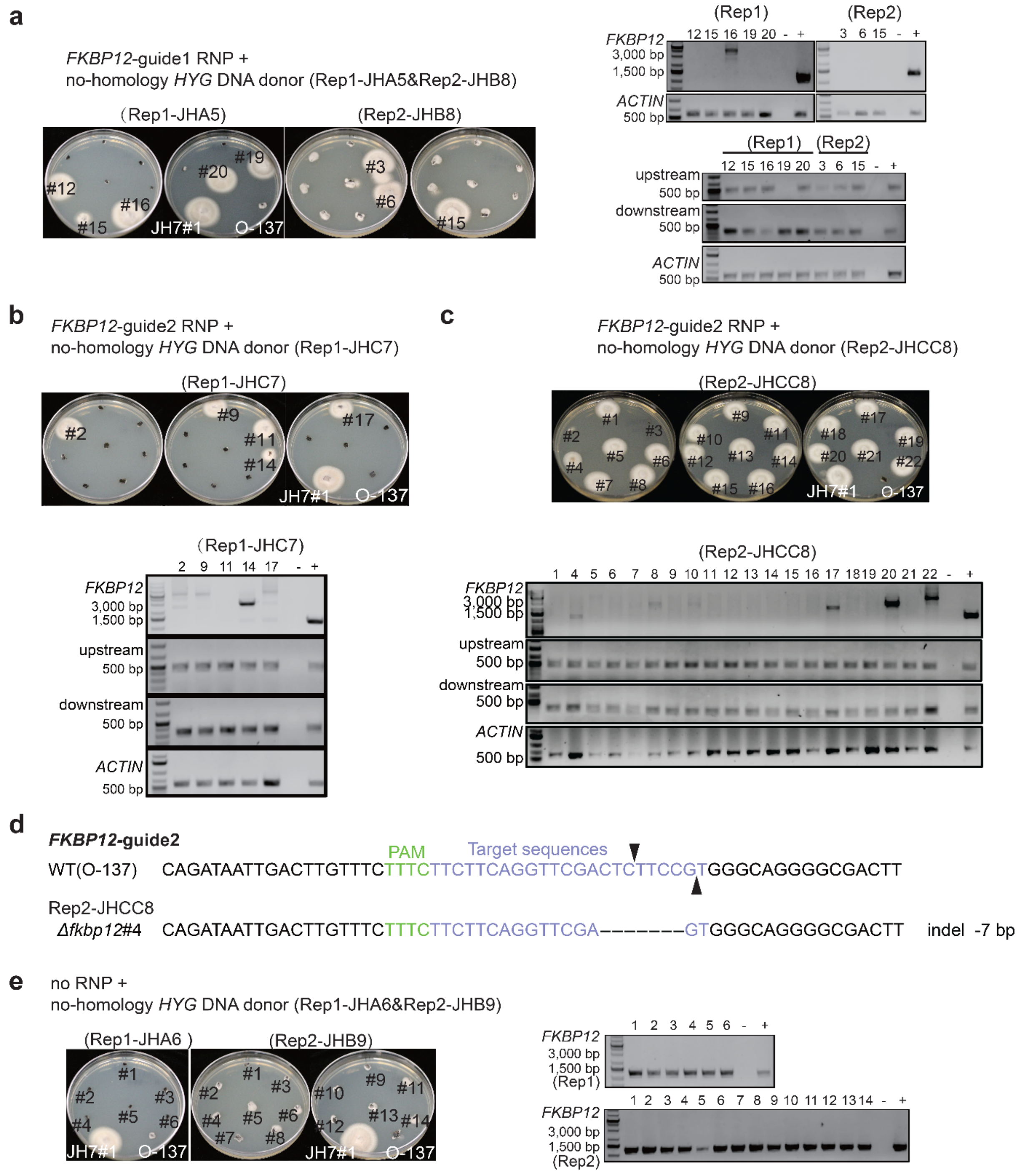

Supplementary Fig. 19 *FKBP12* RNP along with donor DNA generates FK506 insensitive strains.

(a) Hygromycin resistant transformants with *FKBP12*-guide1 RNP along with no-homology *HYG* DNA donor were plated on CM supplemented with 1 μg/mL FK506 to test the drug sensitivity. JH7#1 is an *Δfkbp12* mutant used as a positive control for FK506 insensitivity, O-137 is the wild-type isolate used in the assay. Two independent transformation assays were performed (JHA5 and JHB8). The FK506 insensitive strains were used for further genotyping with primer pairs

spanning *FKBP12* coding sequence, 5' upstream and 3' downstream. *ACTIN* was used as a control for proper DNA extraction. – and + indicate the negative control (water) and positive control (O-137 genomic DNA) for PCR reaction. Similar layout but for the hygromycin resistant transformants with *FKBP12*-guide2 RNP along with no-homology *HYG* DNA donor from two biological replications (JHC7 and JHCC8) were showed in (b and c). (d) The mutational profile of the INDEL mutant from *FKBP12*-guide2 RNP targeting assay is presented. (e) Hygromycin resistant transformants with no-homology *HYG* DNA donor alone from two biological replications were plated for phenotyping and further genotyping with the similar strategy used in (a, b, and c).

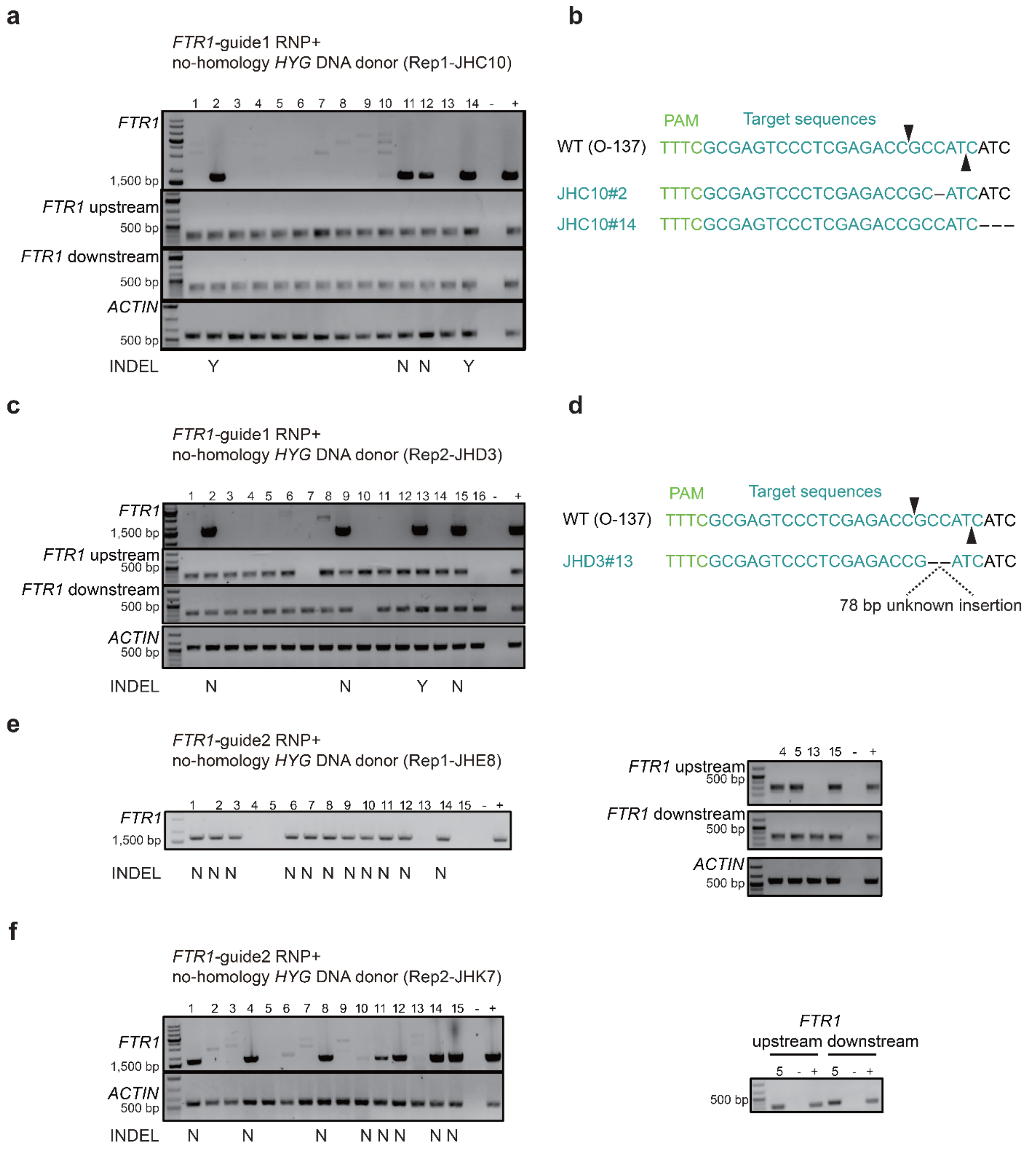

Supplementary Fig. 20 Genotyping for transformants from *FTR1* RNPs and no-homology *HYG* DNA donor.

(a) PCR genotyping results for hygromycin resistant transformants from *FTR1*-guide1 RNP with no-homology *HYG* DNA donor. The wild-type-like PCR products were purified and Sanger sequenced to detect the potential INDELs. INDEL N indicates there were no INDELs observed after sequencing, otherwise labelled as INDEL Y. (b) The sequence alignment between wild-type and INDEL strains from (a). Biological replication result for same guide1 is placed in (c) and (d). (e and f) Replicated PCR genotyping results for hygromycin resistant transformants from *FTR1*-

guide2 RNP with no-homology *HYG* DNA donor. – and + indicate the negative control (water) and positive control (O-137 genomic DNA) for PCR reaction. Two independent transformations and further genotyping were performed for each guide.

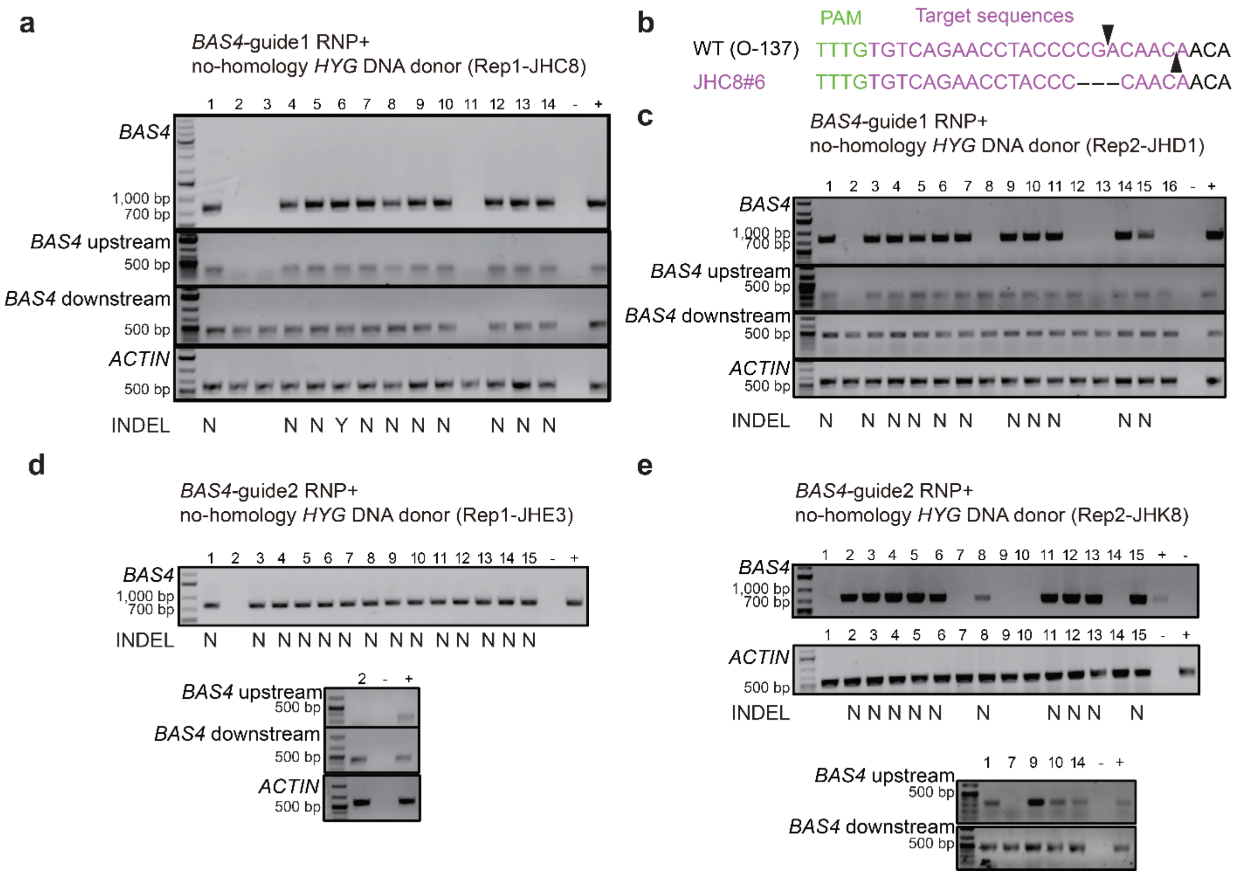

Supplementary Fig. 21 Genotyping for transformants from *BAS4* RNPs and no-homology *HYG* DNA donor.

(a) PCR genotyping results for hygromycin resistant transformants from *BAS4*-guide1 RNP and no-homology *HYG* DNA donor. The wild-type-like PCR products were purified and Sanger sequenced to detect the potential INDEL. INDEL N indicates there were no INDELs observed after sequencing, otherwise labelled as INDEL Y. (b) The sequence alignment between wild-type and INDEL strains from (a). Independent replication for same guide1 were shown in (c). (d and e) Replicated PCR genotyping results for hygromycin resistant transformants from *BAS4*-guide2 RNP and no-homology *HYG* DNA donor. – and + indicate the negative control (water) and positive control (O-137 genomic DNA) for PCR reaction. Two independent transformations and further genotyping were performed for each guide.

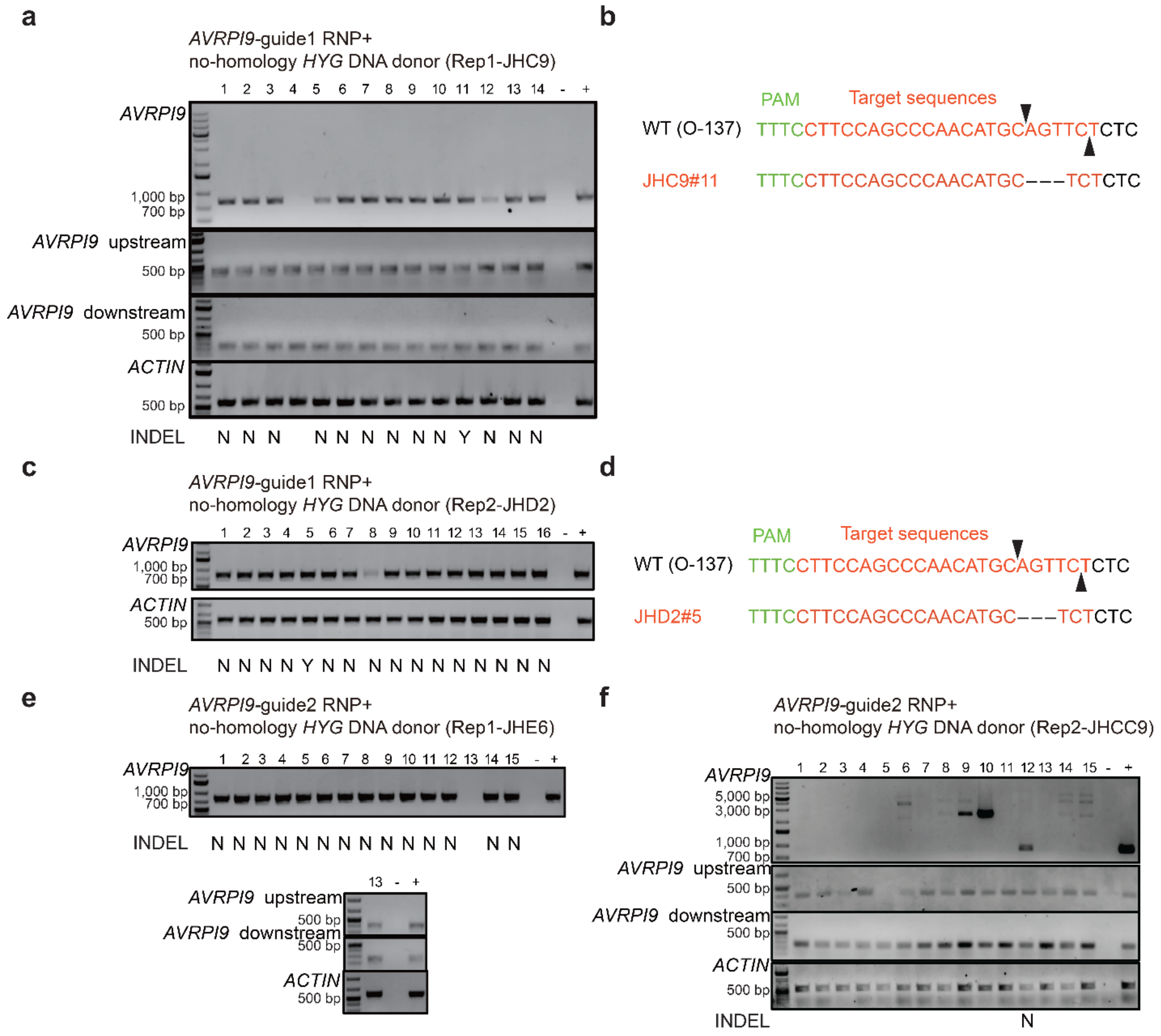

Supplementary Fig. 22 Genotyping for transformants from *AVRPI9* RNPs and no-homology *HYG* DNA donor.
(a) PCR genotyping results for hygromycin resistant transformants from *AVRPI9*-guide1 RNP and no-homology *HYG* DNA donor. The wild-type-like PCR products were purified and Sanger sequenced to detect the potential INDELs. INDEL N indicates there were no INDELs observed after sequencing, otherwise labelled as INDEL Y. (b) The sequence alignment between wild-type and INDEL strains from (a). Biological replication result for same guide1 were placed in (c) and (d). (e and f) Replicated PCR genotyping results for hygromycin resistant transformants from *AVRPI9*-guide2 RNP and no-homology *HYG* DNA donor. – and + indicate the negative control (water) and positive control (O-137 genomic DNA) for PCR reaction. Two independent transformations and further genotyping were performed for each guide.

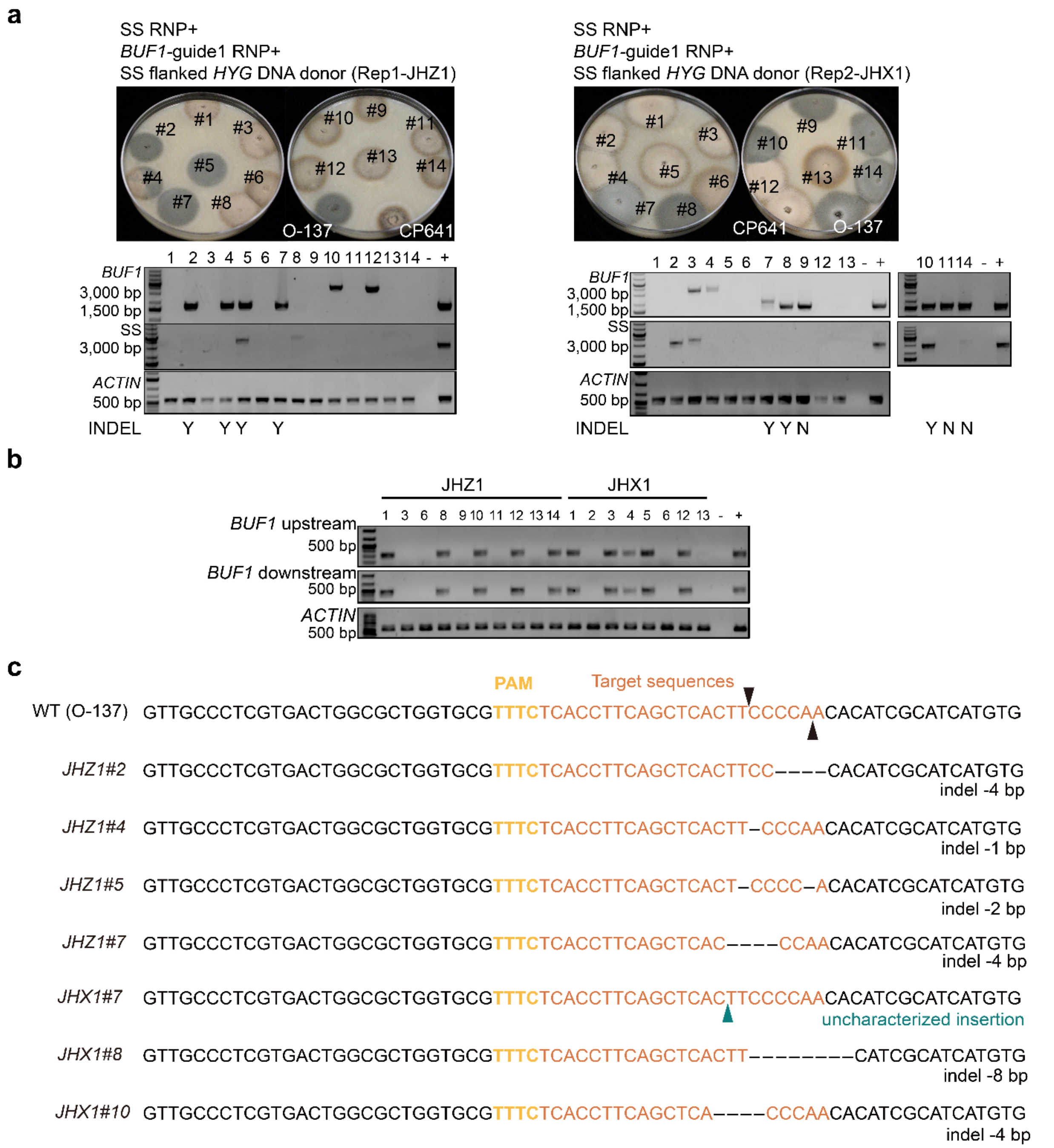

Supplementary Fig. 23 Genotyping and phenotyping of *BUF1* transformants from the second-site targeting assay.

(a) Hygromycin resistant transformants were plated on OTA for phenotyping the mycelial color change. DNA was extracted and used for genotyping with primary-site (*BUF1*), second-site (SS) and *ACTIN* (control). The wild-type-like PCR products from primary-site were purified and Sanger sequenced to detect the potential INDELs. INDEL N indicates there were no INDELs observed after sequencing, otherwise labelled as INDEL Y. CP641 derived from O-137, is positive strain for showing buff phenotype, O-137 is the wild-type isolate used in these assays. The assays were repeated twice independently (rep1-JHZ1 and rep2-JHX1). (b) All the PCR

negative strains and some PCR positive strains from *BUF1* locus in (a) were further characterized with *BUF1* 5' upstream and 3' downstream primers. – and + indicate the negative control (water) and positive control (O-137 genomic DNA) for PCR reaction. (c) The sequence alignment between wild-type and the strains with INDEL at *BUF1* targeting region identified from (a) is showed.

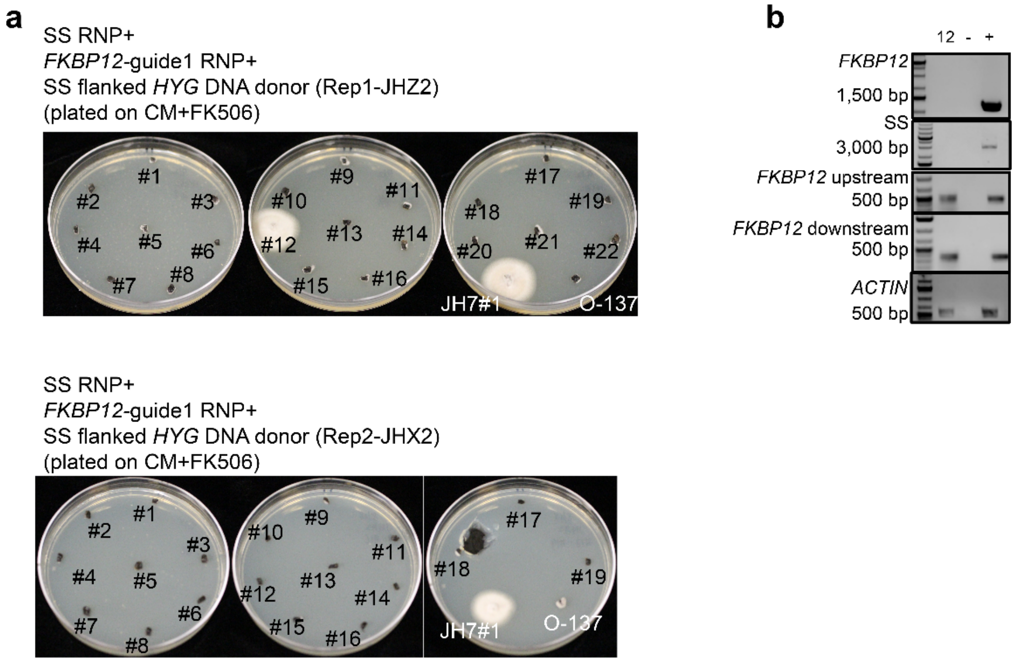

Supplementary Fig. 24 Genotyping and phenotyping of *FKBP12* transformants from the second-site targeting assay.

(a) Hygromycin resistant transformants were plated on CM+FK506 for phenotyping the FK506 resistance. The assays were repeated twice (rep1-JHZ2 and rep2-JHX2). JH7#1 derived from O-137 is a control strain for FK506 resistance, O-137 is the wild-type used in these assays. (b) DNA from the FK506 resistant strain (JHZ2#12) was extracted and used for genotyping with primary-site (*FKBP12*), second-site (SS), *FKBP12* 5' upstream/3' downstream and *ACTIN* (control). – and + indicate the negative control (water) and positive control (O-137 genomic DNA) for PCR reaction. Two independent transformations and further genotyping were performed.

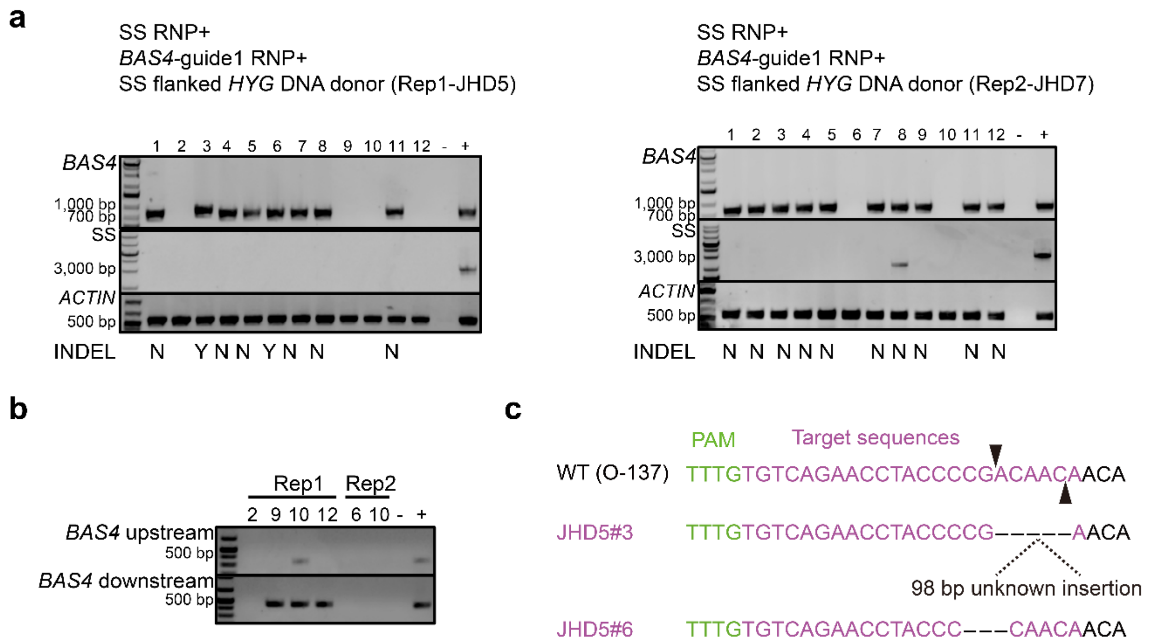

Supplementary Fig. 25 Genotyping of *BAS4* transformants from the second-site targeting assay.

(a) Hygromycin resistant transformants were used for DNA extraction and further genotyping with primary-site (*BAS4*), second-site (SS) and *ACTIN* (control). The wild-type-like PCR products from primary-site were purified and Sanger sequenced to detect the potential INDELs. INDEL N indicates there were no INDELs observed after sequencing, otherwise labelled as INDEL Y. The assays were repeated twice (rep1-JHD5 and rep2-JHD7). (b) PCR negative strains from *BAS4* locus in (a) were further characterized with *BAS4* 5' upstream and 3' downstream primers. – and + indicate the negative control (water) and positive control (O-137 genomic DNA) for PCR reaction. (c) The sequence alignment between wild-type and the strains with INDEL at *BAS4* targeting region identified from (a) is showed.

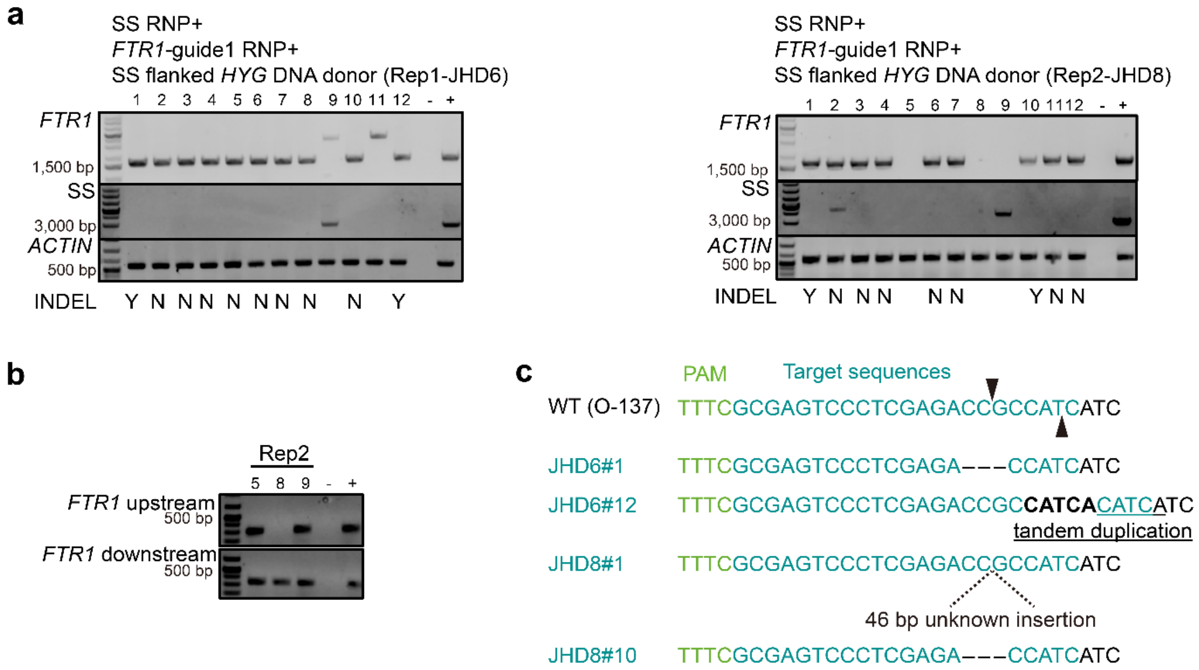

Supplementary Fig. 26 Genotyping of *FTR1* transformants from the second-site targeting assay.

(a) Hygromycin resistant transformants were used for DNA extraction and further genotyping with primary-site (*FTR1*), second-site (SS) and *ACTIN* (control). The wild-type-like PCR products from primary-site were purified and Sanger sequenced to detect the INDEL. INDEL N indicated there were no INDELs observed after sequencing, otherwise labeled as INDEL Y. The assays were repeated twice (rep1-JHD6 and rep2-JHD8). (b) PCR negative strains from *FTR1* locus in (a) were further characterized with *FTR1* 5' upstream and 3' downstream primers. – and + indicate the negative control (water) and positive control (O-137 genomic DNA) for PCR reaction. (c) The sequence alignment between wild-type and the strains with INDEL at *FTR1* targeting region identified from (a) is showed. Underlined letters indicated the potential template for tandem duplication.

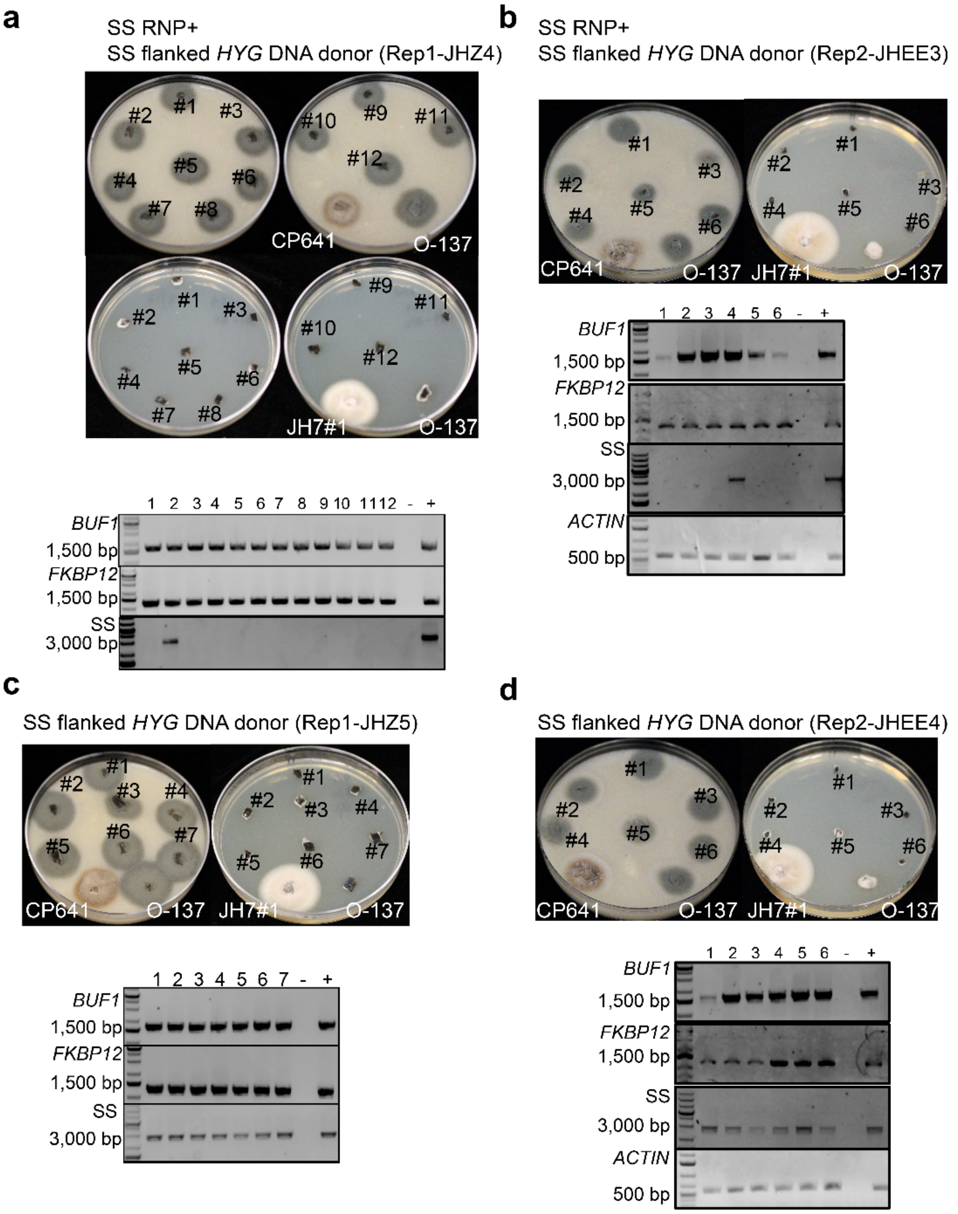

Supplementary Fig. 27 The second-site RNP along with the second-site donor or donor alone serve as control for the second-site targeting assay.

(a and b) Replicated hygromycin resistant transformants from Second-site RNP along with second-site (SS) donor were plated on OTA and CM+FK506 for phenotyping, and further genotyping with primary-site (*BUF1* and *FKBP12*), second-site (SS). (c and d) Replicated phenotyping and genotyping results for hygromycin resistant transformants from SS donor alone are presented, with similar layout as (a). Two independent transformations and further genotyping were performed for each control.

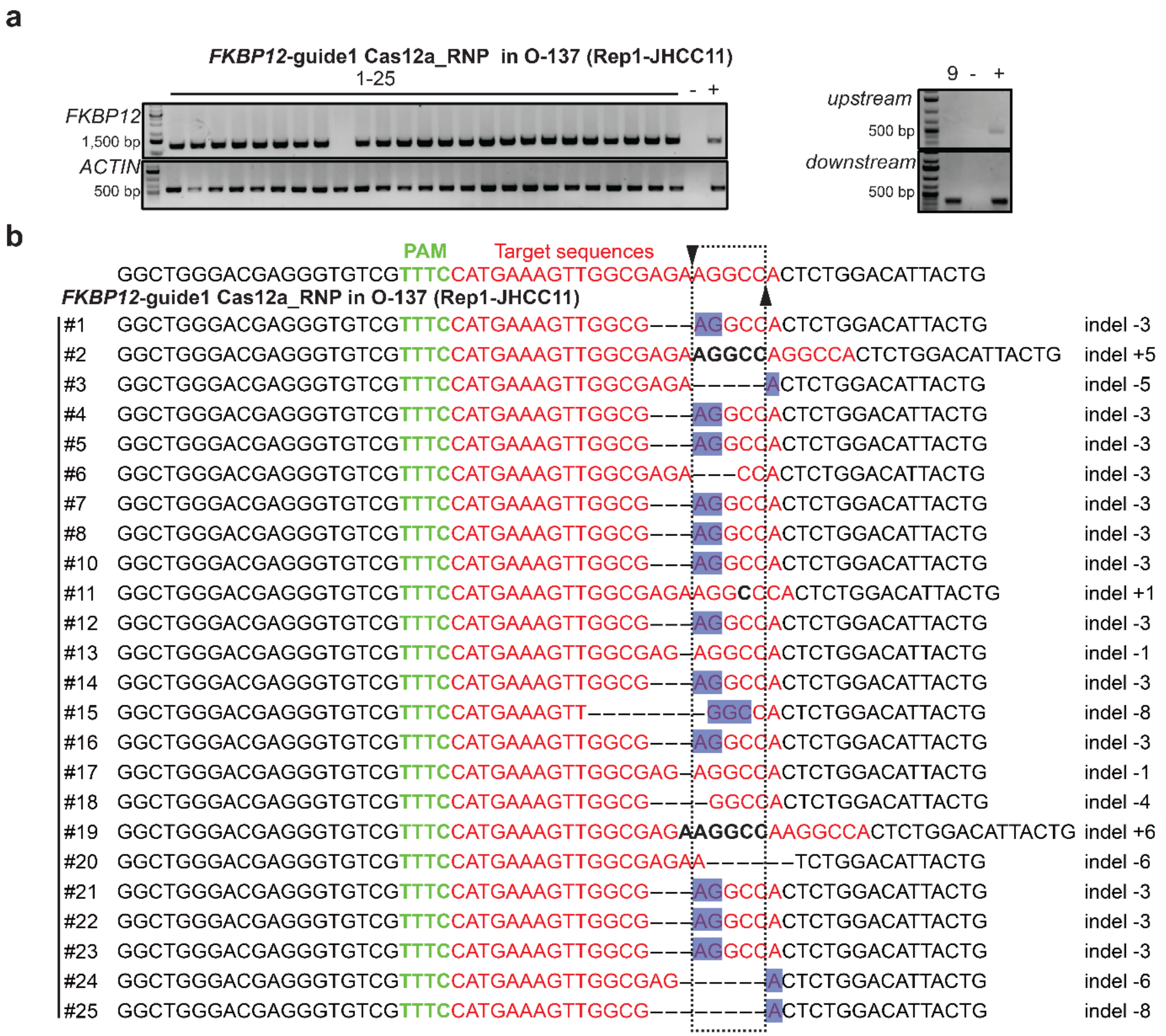

Supplementary Fig. 28 Genotyping of Cas12a *FKBP12*-guide1 RNP edited FK506^R transformants from O-137 background in the absence of DNA donor (replication 1).
(a) FK506 resistant transformants from the first biological replicate were used for DNA extraction and further genotyping with *FKBP12* and *ACTIN* (control). The transformants with PCR negative from *FKBP12* genotyping result were further amplified with *FKBP12* 5'upstream and 3'downstream primer pairs. – and + indicate the negative control (water) and positive control (O-137 genomic DNA) for PCR reaction. (b) The sequence alignment between wild-type and the FK506 resistant strains with PCR positive outcomes. Green and red letters indicate the PAM and target sequences. Black triangles and dash lines around the target sequences highlight the potential Cas12a cut site. Bold letters, dash lines, and blue boxes within the sequences of each individual transformants (#1-#25) indicate the small insertions, deletions, and microhomology flanking the repair.

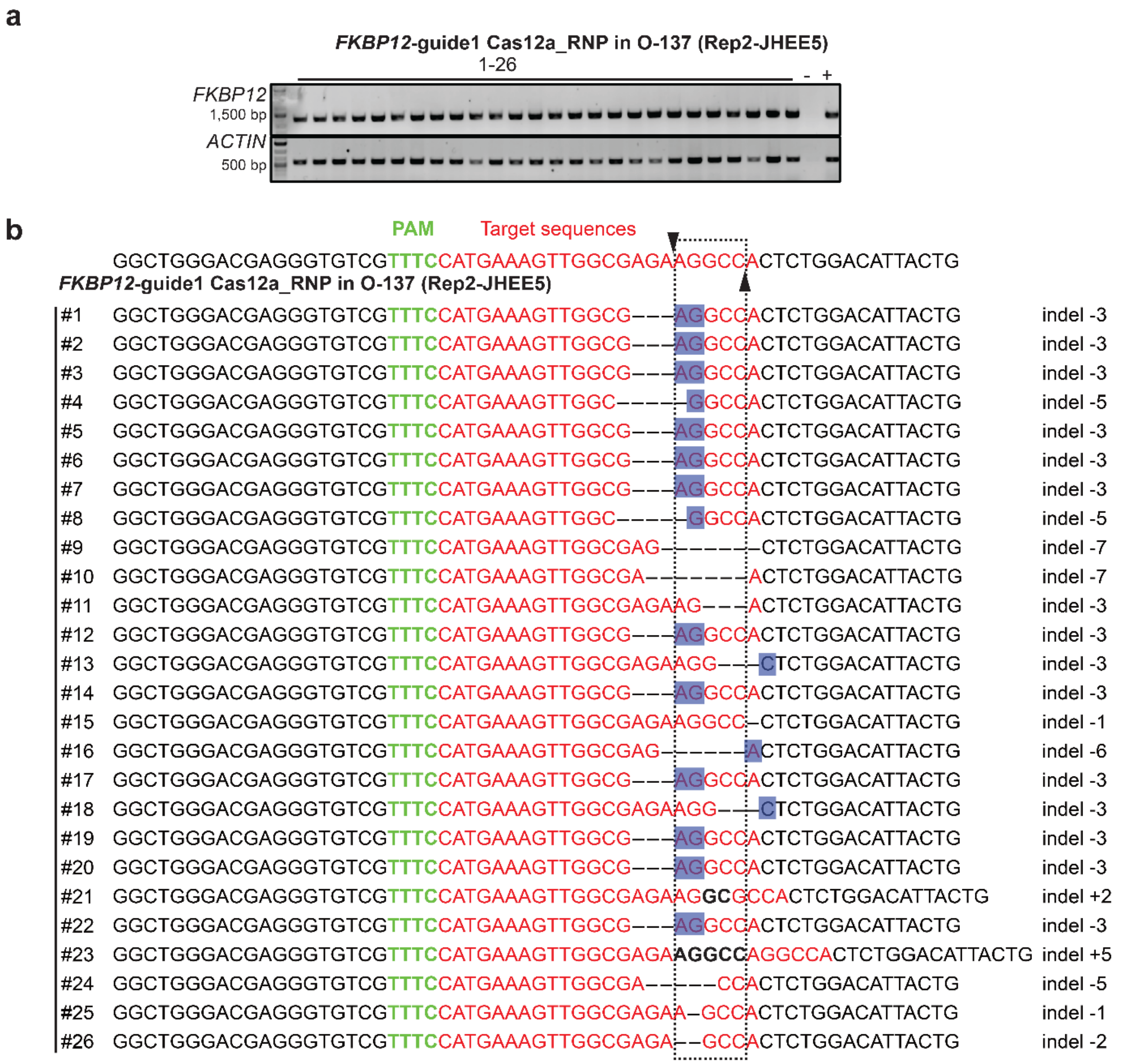

Supplementary Fig. 29 Genotyping of Cas12a *FKBP12*-guide1 RNP edited FK506^{R} transformants from O-137 background in the absence of DNA donor (replication 2).
(a) Similar PCR genotyping strategy as described in Fig. S28a was used for the FK506 resistant transformants from the second biological replicate. (b) Similar sequence alignment layout as shown in Fig. S28b was displayed for the FK506 resistant transformants (#1-#29) from the second biological replicate.

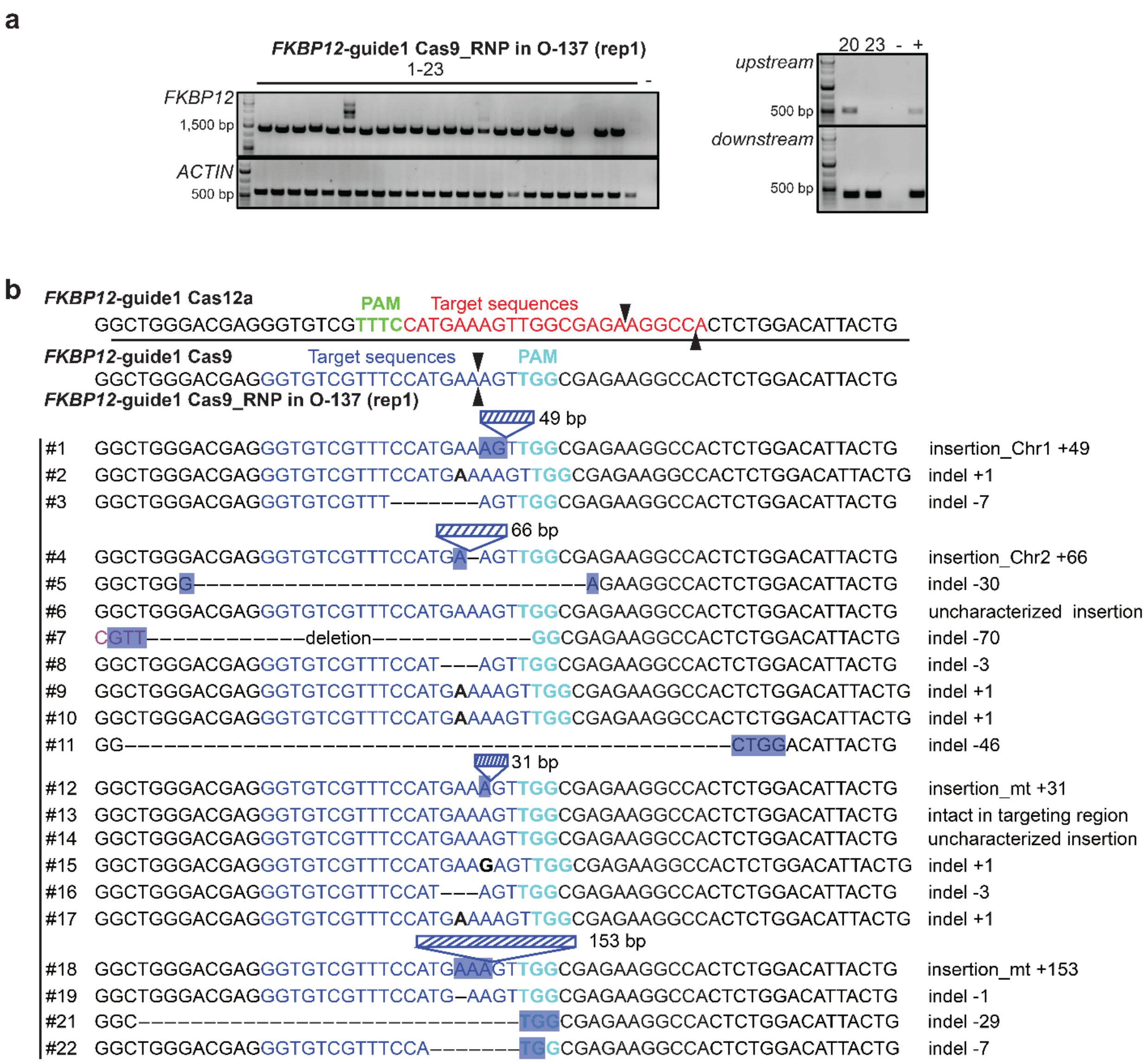

Supplementary Fig. 30 Genotyping of Cas9 *FKBP12*-guide1 RNP edited FK506^{R} transformants from O-137 background in the absence of DNA donor (replication 1).

(a) FK506 resistant transformants from the first biological replicate were used for DNA extraction and further genotyping with *FKBP12* and *ACTIN* (control). The transformants with PCR negative from *FKBP12* genotyping result were further amplified with *FKBP12* 5'upstream and 3'downstream primer pairs. – indicates the negative control (water) for PCR reaction. (b) The sequence alignment between wild-type and the FK506 resistant strains with PCR positive outcomes. Green and red letters indicate the PAM and target sequences for Cas12a, light and dark blue highlight the PAM and target sequences for Cas9. Black triangles around the target sequences highlight the potential Cas12a and Cas9 cut site. Bold letters, dash lines, and blue boxes within the sequences of each individual transformants (#1-#22) indicate the small insertions, deletions, and microhomology flanking the repair. If the deletion is larger than the sequences presented in the wild-type, the new end sequences at the repair junction are labelled with purple letters. Insertions from either other part of chromosome or mitochondrion

were highlighted as blue rectangle. Note: Exact sequences in #6 and #14 transformants had not been fully identified due to the quality of Sanger sequencing outcome. Additionally, no DNA mutation has been found in transformant #13.

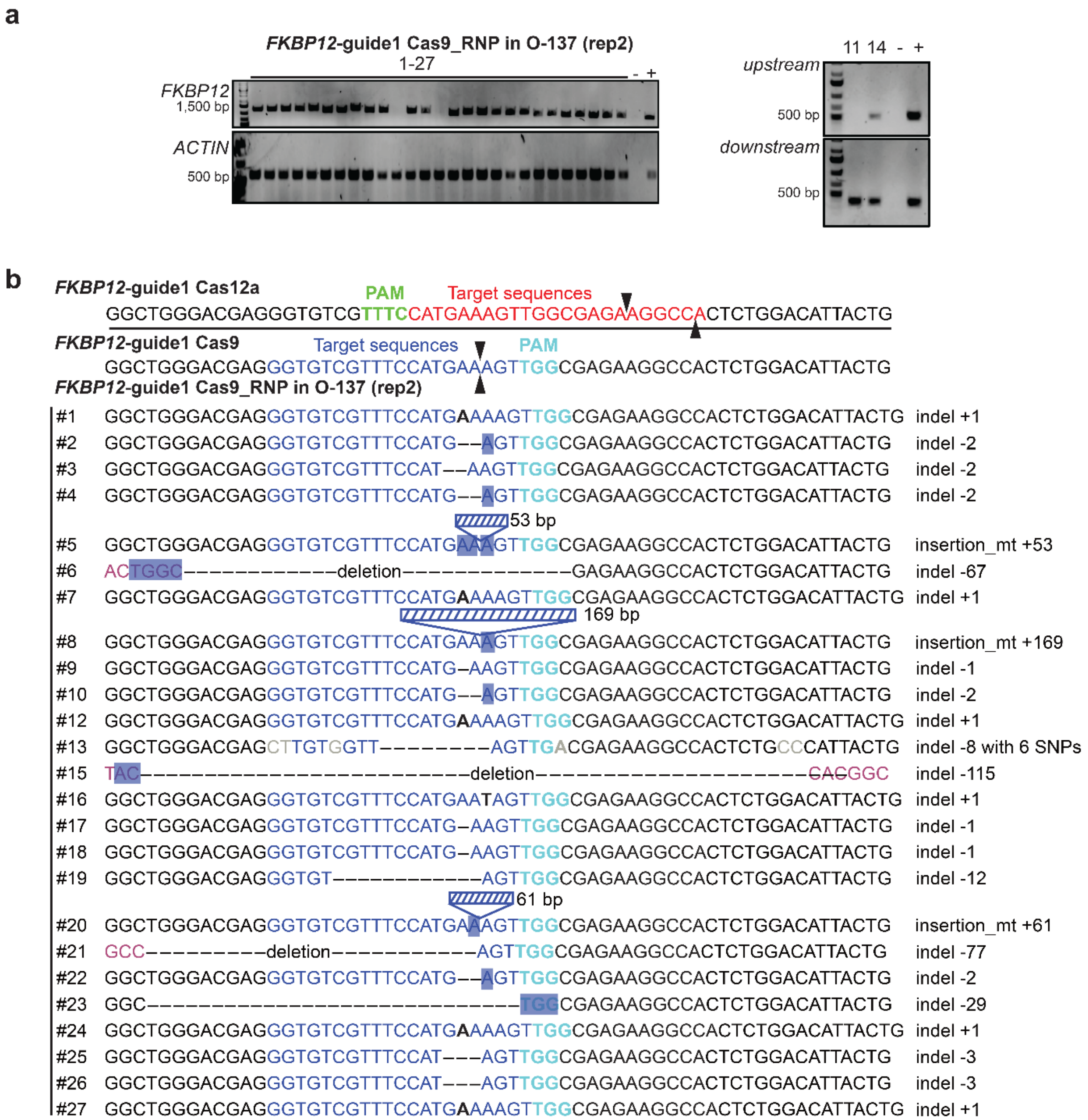

Supplementary Fig. 31 Genotyping of Cas9 *FKBP12*-guide1 RNP edited FK506^{R} transformants from O-137 background in the absence of DNA donor (replication 2).

(a) Similar PCR genotyping strategy as described in Fig. S30a was used for the FK506 resistant transformants from the second biological replicate. (b) Similar sequence alignment layout as shown in Fig. S30b was displayed for the FK506 resistant transformants (#1-#27) from the second biological replicate.

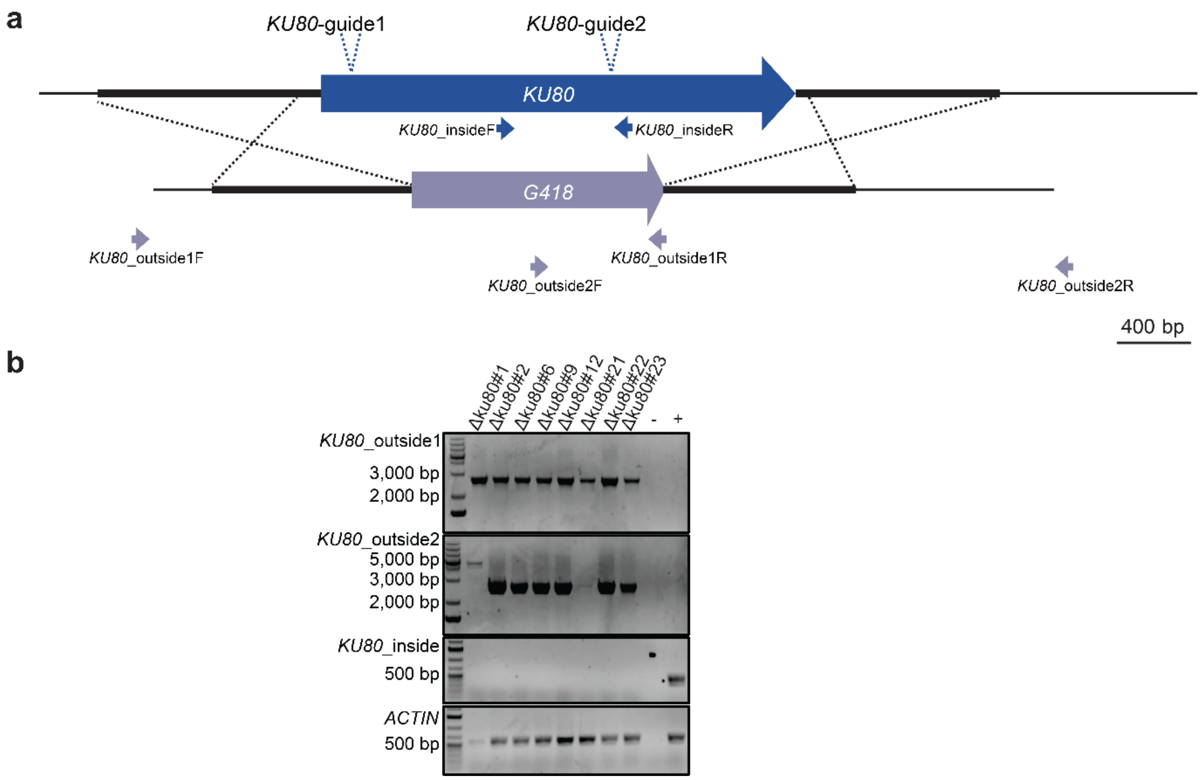

Supplementary Fig. 32 Cas12a along with long-flanking *G418* DNA donor was used for deleting *KU80*.

(a) Schematic diagram of Cas12a RNP mediated *KU80* gene deletion. Two distinct Cas12a gRNAs (i.e., *KU80*-guide1 and *KU80*-guide2) were used for generating DSB within *KU80* gene body. A long-flanking *G418* DNA donor (i.e., 1,078 bp and 1,040 bp sequences homologous to the *KU80* locus was added) was constructed to promote the integration. The deletion of *KU80* was confirmed with KU80_insideF/R primer pair, while KU80_outside1F/R and KU80_outside2F/R were utilized for test the correct insertion of DNA donor. (b) Detailed genotyping results by using the primer pairs as described in (a) were presented. *ACTIN* was used as a control for proper DNA extraction. – and + indicate the negative control (water) and positive control (O-137 genomic DNA) for PCR reaction. The genotyping was repeated twice independently with similar results.

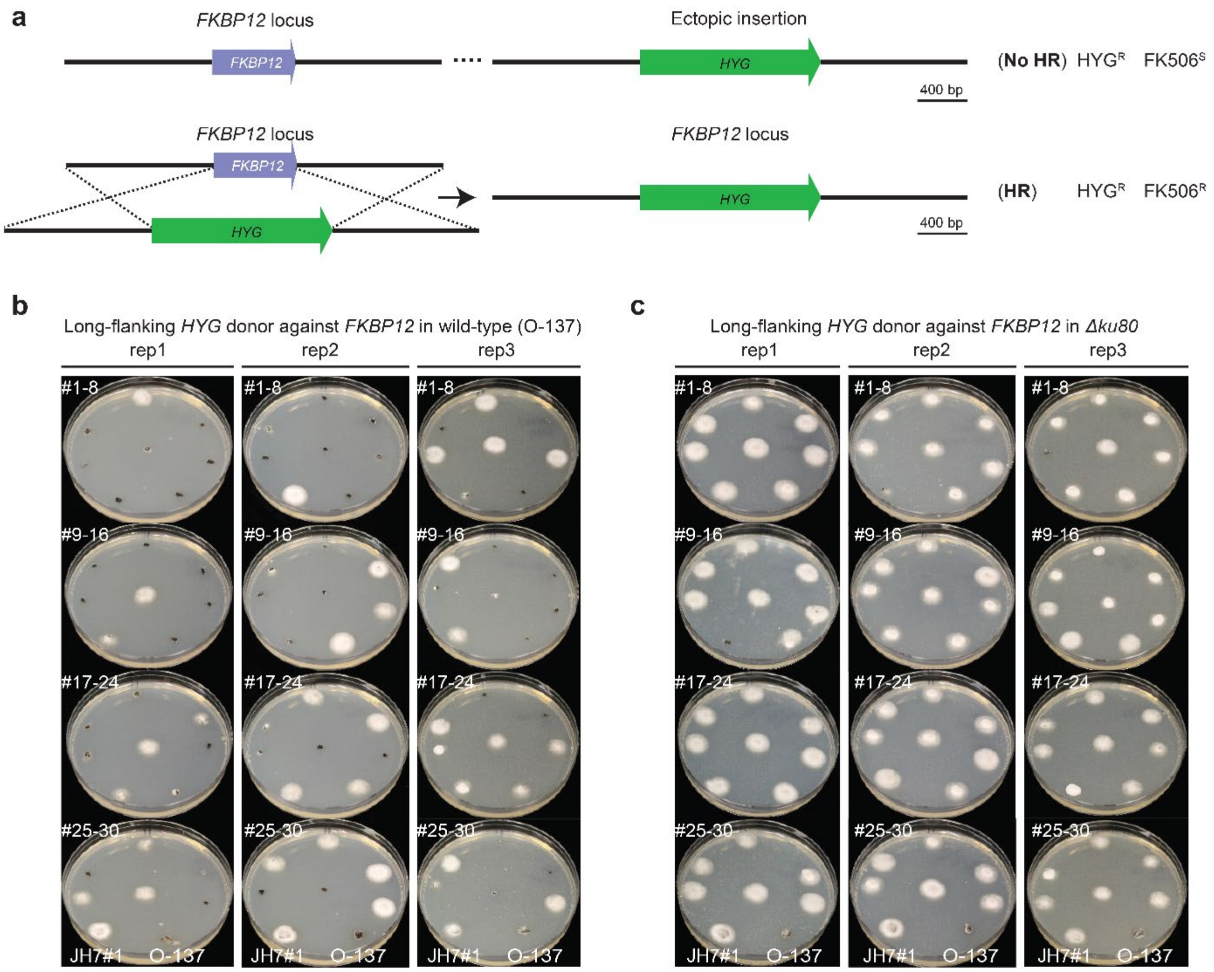

Supplementary Fig. 33 Increased HR frequency by using *Δku80*.
(a) Schematic diagram of the assay testing the frequency of HR in wild-type and *Δku80*. A long-flanking *HYG* DNA donor against *FKBP12* (i.e., 1,173 bp and 1,165 bp sequences homologous to the *FKBP12* locus was added) was used for measuring the rate of HR. The transformants with both hygromycin resistance (HYG^R) and FK506 resistance ($FK506^R$) are the products of HR repair in *FKBP12* locus. If the transformants are hygromycin resistant (HYG^R) but sensitive to FK506 ($FK506^S$), it suggests the ectopic insertion of DNA donor. (b) Following transformations, hygromycin resistant transformants from wild-type (left panel) and *Δku80* (right panel) were plated to CM supplemented with FK506. JH7#1 is an *Δfkbp12* mutant used as a positive control for FK506 insensitivity, O-137 is the wild-type isolate used in the assay. Total 90 transformants (3 biological replicates) from either wild-type or *Δku80* were phenotyped independently. Photos were taken 3 days after plating.

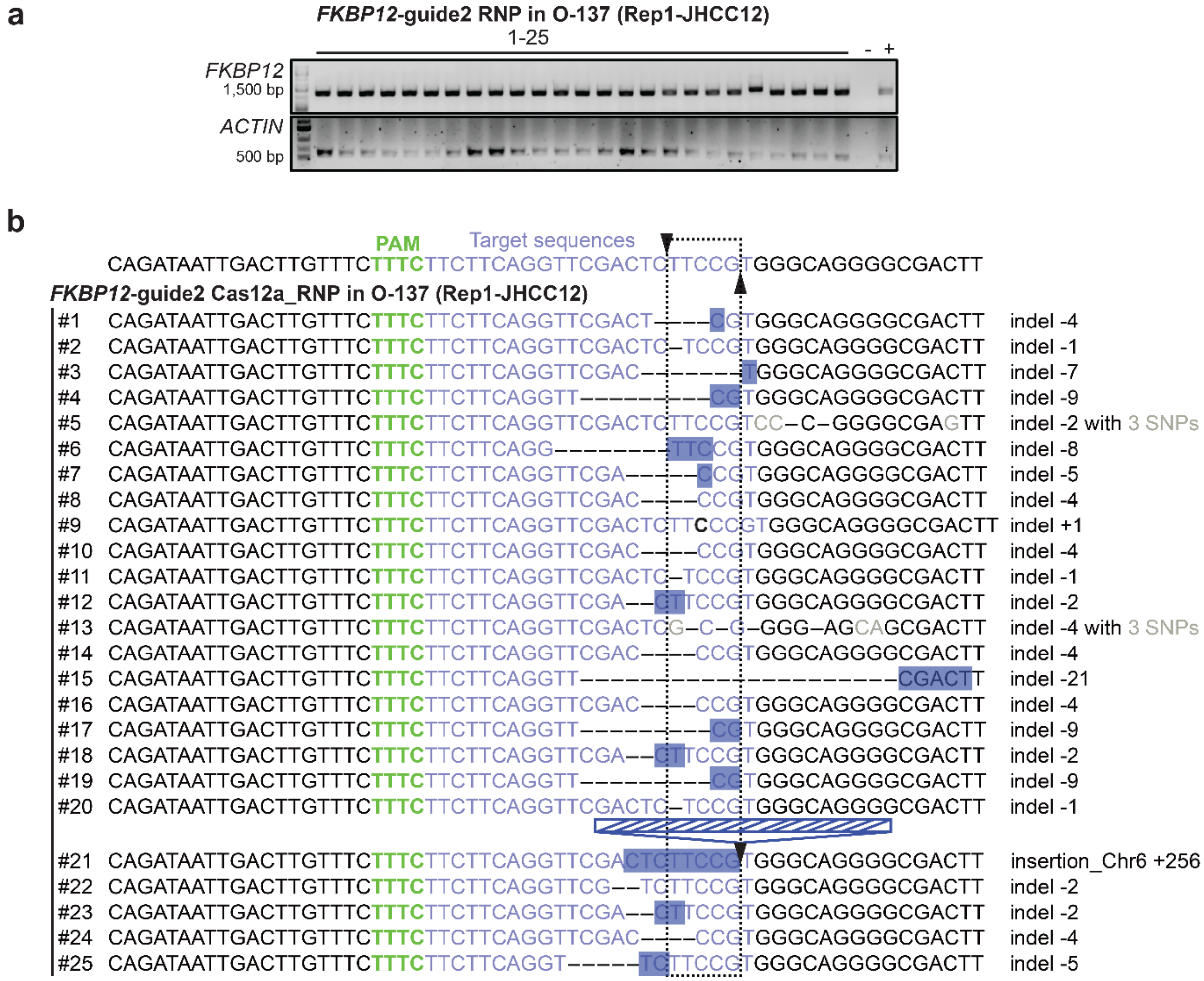

Supplementary Fig. 34 Genotyping of Cas12a *FKBP12*-guide2 RNP edited FK506^{R} transformants from O-137 background in the absence of DNA donor (replication 1).

(a) FK506 resistant transformants from the first biological replicate were used for DNA extraction and further genotyping with *FKBP12* and *ACTIN* (control). – and + indicate the negative control (water) and positive control (O-137 genomic DNA) for PCR reaction. (b) The sequence alignment between wild-type and the FK506 resistant strains with PCR positive outcomes. Green and blue letters indicate the PAM and target sequences. Black triangles and dash lines around the target sequences highlight the potential Cas12a cut site. Bold letters, dash lines, and blue boxes within the sequences of each individual transformants (#1-#25) indicate the small insertions, deletions, and microhomology flanking the repair. Grey letters highlight the SNP. Insertions from either other part of chromosome or mitochondrion were highlighted as blue rectangle.

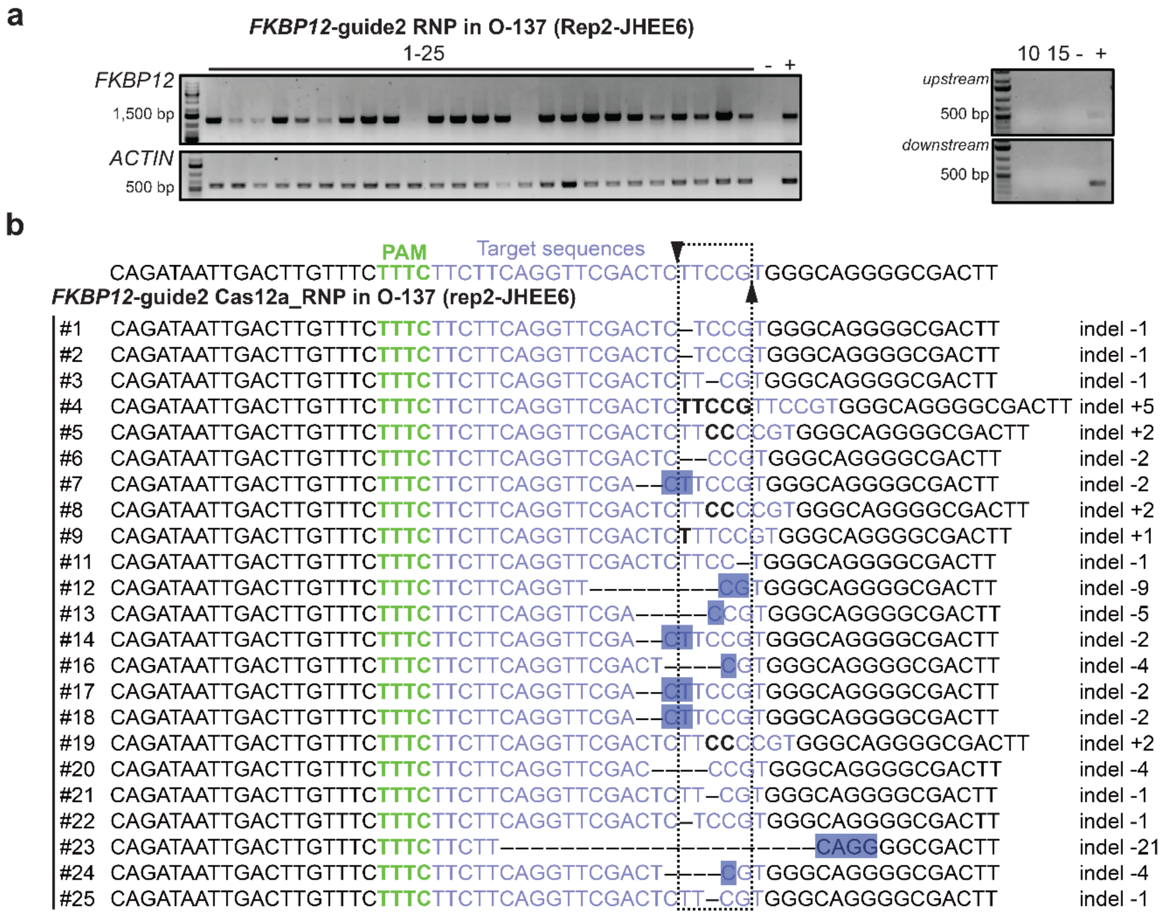

Supplementary Fig. 35 Genotyping of Cas12a *FKBP12*-guide2 RNP edited FK506^R transformants from O-137 background in the absence of DNA donor (replication 2).
(a) Similar PCR genotyping strategy as described in Fig. S34a was used for the FK506 resistant transformants from the second biological replicate. The transformants with PCR negative *FKBP12* genotyping result were further amplified with *FKBP12* 5'upstream and 3'downstream primer pairs. (b) Similar sequence alignment layout as shown in Fig. S34b was displayed for the FK506 resistant transformants (#1-#25) from the second biological replicate.

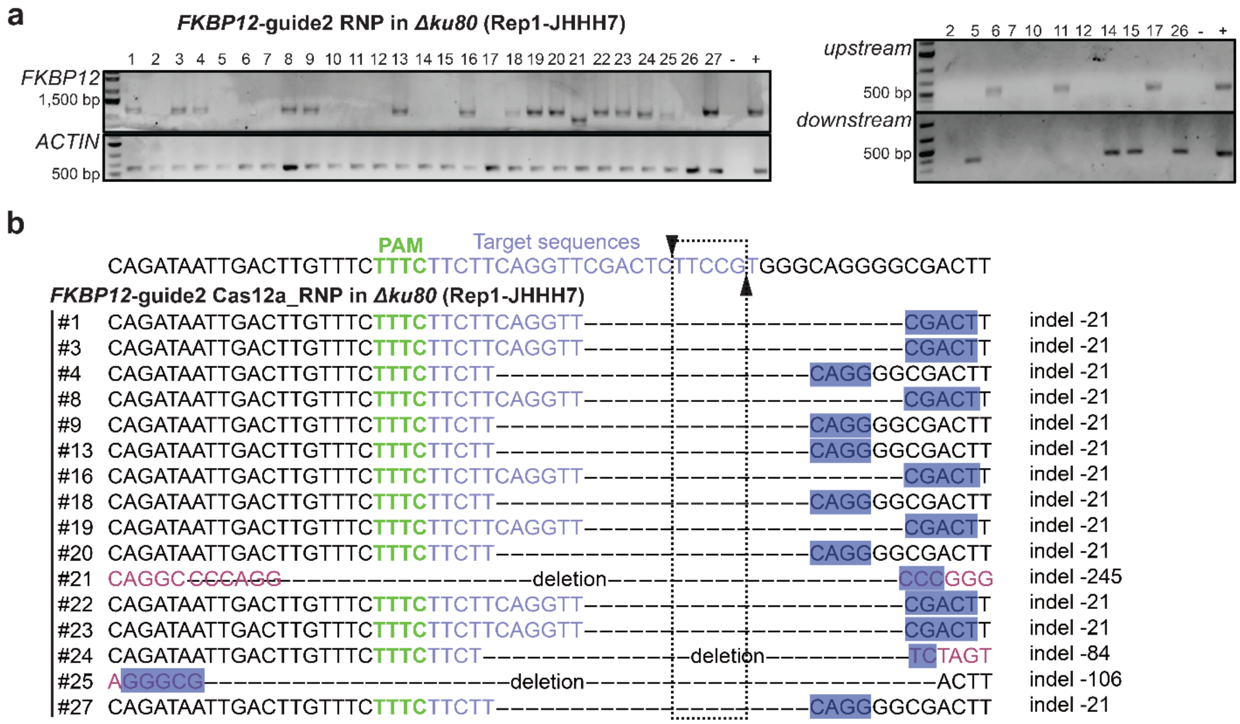

Supplementary Fig. 36 Genotyping of Cas12a *FKBP12*-guide2 RNP edited FK506^{R} transformants from *Δku80* background in the absence of DNA donor (replication 1).
(a) FK506 resistant transformants from the first biological replicate were used for DNA extraction and further genotyping with *FKBP12* and *ACTIN* (control). The transformants with PCR negative from *FKBP12* genotyping result were further amplified with *FKBP12* 5'upstream and 3'downstream primer pairs. – and + indicate the negative control (water) and positive control (O-137 genomic DNA) for PCR reaction. (b) The sequence alignment between wild-type and the FK506 resistant strains with PCR positive outcomes. Green and blue letters indicate the PAM and target sequences. Black triangles and dash lines around the target sequences highlight the potential Cas12a cut site. Dash lines, and blue boxes within the sequences of each individual indicate deletions, and microhomology flanking the repair. If the deletion is larger than the sequences presented in the wild-type, the new end sequences at the repair junction are labelled with purple letters.

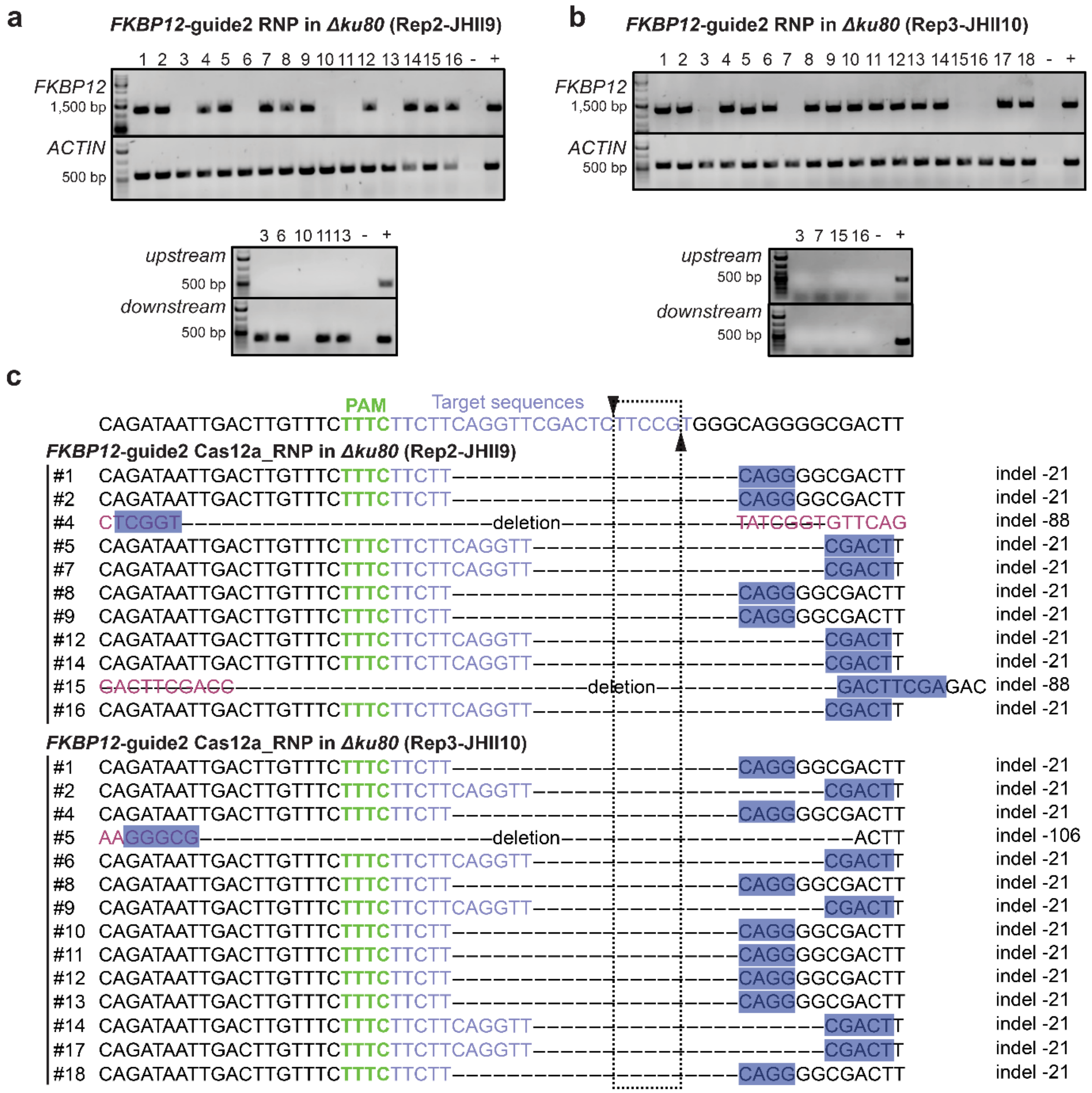

Supplementary Fig. 37 Genotyping of Cas12a *FKBP12*-guide2 RNP edited FK506^{R} transformants from *Δku80* background in the absence of DNA donor (replication 2 and 3). (a) Similar PCR genotyping strategy as described in Fig. S36a was used for the FK506 resistant transformants from the second and third biological replicates. (b) Similar sequence alignment layout as shown in Fig. S36b was displayed for the FK506 resistant transformants from the second and third biological replicates.

a

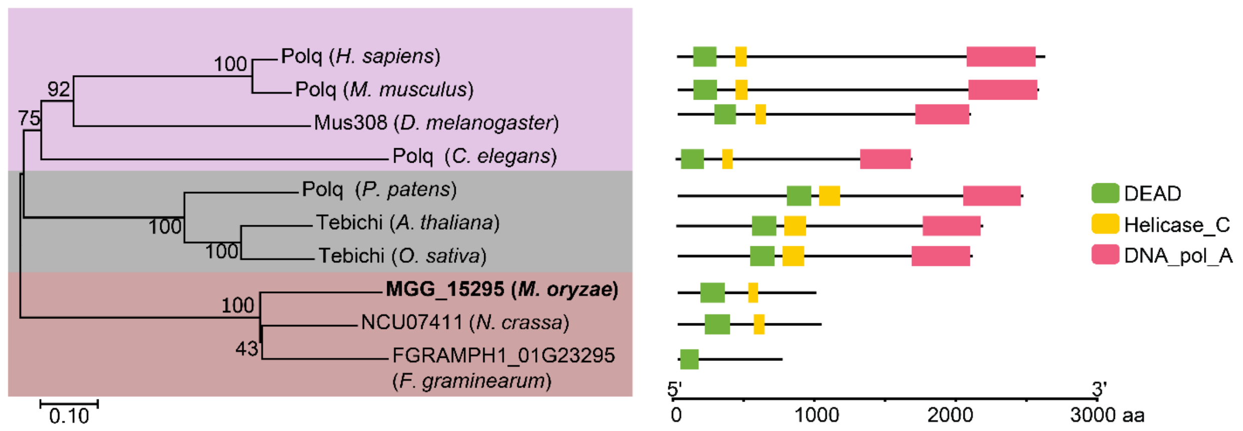

b

	Polq (*Hs*)	Polq (*Ms*)	Polq (*Dm*)	Tebichi (*At*)	Tebichi (*Os*)	Polq (*Pp*)	Mus308 (*Ce*)	Polq (*Nc*)	Polq (*Mo*)	Polq (*Fg*)	
Polq (*Hs*)	100%/ 100%	99%/ 72%	64%/ 43%	56%/ 41%	56%/ 41%	56%/ 41%	51%/ 33%	30%/ 33%	31%/ 31%	27%/ 30%	coverage/ identity

Supplementary Fig. 38 Phylogenetic and sequence similarity analysis of *POLQ* homologs. (a) Neighbor-joining tree of selected *POLQ* homologs generated by MEGA X with 1000 bootstrap replications. Domain distribution of each protein is shown in the right panel. (b) Homology matrix analysis of selected *POLQ* in different organisms. Numbers indicate protein coverage and identity.

Supplementary Tables

Supplementary Table 1 Summary for flanked DNA donor assay with *BUF1*

Isolate	gRNA used	Selection	Donor DNA	# of HYGR transformants	# of non-buff transformants	# of buff mutants	# of PCR validated transformants	# of PCR confirmed buf1 mutants	# of PCR-negative buf1 mutants	# of simple insertion buf1 mutants	# of INDEL buf1 mutants	Note	Figures
O-137	*BUF1* -gRNA1	HYG	flanked *HYG* DNA donor 1	10	3	7	10	7	7	0	0	rep1	Fig1c,e,f
O-137	*BUF1* -gRNA1	HYG	flanked *HYG* DNA donor 1	22	1	21	22	22	21	0	1	rep2, JHCC1	Fig1f, and FigS1a,e
O-137	*BUF1* -gRNA1	HYG	flanked *HYG* DNA donor 1	16	1	15	16	15	12	2	1	rep3, JHAA1	Fig1f, and FigS1b,e
O-137	*BUF1* -gRNA2	HYG	flanked *HYG* DNA donor 2	13	6	7	13	7	3	4	0	rep1	Fig1c,e,f
O-137	*BUF1* -gRNA2	HYG	flanked *HYG* DNA donor 2	22	0	22	22	22	22	0	0	rep2, JHCC2	Fig1f, and FigS1c
O-137	*BUF1* -gRNA2	HYG	flanked *HYG* DNA donor 2	22	13	9	22	9	8	0	1	rep3, JHAA2	Fig1f, and FigS1d,e
O-137	N/A	HYG	flanked *HYG* DNA donor 1	2	2	0	2	0	N/A	N/A	N/A	rep1	Fig1f, and FigS2a
O-137	N/A	HYG	flanked *HYG* DNA donor 1	4	4	0	4	0	N/A	N/A	N/A	rep2, JHCC3	Fig1f, and FigS2c
O-137	N/A	HYG	flanked *HYG* DNA donor 1	8	8	0	8	0	N/A	N/A	N/A	rep3, JHAA3	Fig1f, and FigS2e
O-137	N/A	HYG	flanked *HYG* DNA donor 2	6	6	0	6	0	N/A	N/A	N/A	rep1	Fig1f, and FigS2b
O-137	N/A	HYG	flanked *HYG* DNA donor 2	6	6	0	6	0	N/A	N/A	N/A	rep2, JHCC4	Fig1f, and FigS2d
O-137	N/A	HYG	flanked *HYG* DNA donor 2	6	6	0	6	0	N/A	N/A	N/A	rep3, JHAA4	Fig1f, and FigS2f

Isolate	gRNA used	Selection	Donor DNA	# of HYGR transformants	# of non-buff transformants	# of buff mutants	# of PCR validated transformants	# of PCR confirmed buf1 mutants	# of PCR-negative buf1 mutants	# of simple insertion buf1 mutants	# of INDEL buf1 mutants	Note	Figures
Guy11	*BUF1* -gRNA1	HYG	flanked *HYG* DNA donor 1	31	0	31	8	8	6	2	0	rep1	Fig1g and FigS3a,e
Guy11	*BUF1* -gRNA1	HYG	flanked *HYG* DNA donor 1	22	0	22	22	22	21	1	0	rep2, JHBB1	Fig1g and FigS3g
Guy11	*BUF1* -gRNA2	HYG	flanked *HYG* DNA donor 2	30	1	29	10	9	6	3	0	rep1	Fig1g and FigS3c,f
Guy11	*BUF1* -gRNA2	HYG	flanked *HYG* DNA donor 2	22	1	21	22	21	20	1	0	rep2, JHBB2	Fig1g and FigS3h
Guy11	N/A	HYG	flanked *HYG* DNA donor 1	5	5	0	5	0	N/A	N/A	N/A	rep1	Fig1g and FigS3b,e
Guy11	N/A	HYG	flanked *HYG* DNA donor 1	4	4	0	4	0	N/A	N/A	N/A	rep2, JHBB3	Fig1g and FigS3i
Guy11	N/A	HYG	flanked *HYG* DNA donor 2	18	18	0	8	0	N/A	N/A	N/A	rep1	Fig1g and FigS3d,f
Guy11	N/A	HYG	flanked *HYG* DNA donor 2	3	3	0	3	0	N/A	N/A	N/A	rep2, JHBB4	Fig1g and FigS3j

N/A=not applicable; HYGR=hygromycin resistant; rep=replication

Supplementary Table 2 Summary for no-homology DNA donor assay with *BUF1*

raw data for no-homology assay targeting *BUF1* with hygromycin selection

Isolate	gRNA used	Selection	Donor DNA	# of HYGR transformants	# of non-buff transformants	# of buff mutants	# of PCR validated transformants	# of PCR confirmed buf1 mutants	# of simple insertion buf1 mutants	# of INDEL buf1 mutants	# of PCR-negative buf1 mutants	# of large deletion buf1 mutants	# of deletion plus insertion buf1 mutants	# of large insertion buf1 mutants	Note	Figures
O-137	*BUF1* -gRNA1	HYG	no-homology *HYG* DNA donor	14	8	6	14	7	3	1	3	1	0	2	rep1	Fig2b,c,f, FigS7a and FigS17b
O-137	*BUF1* -gRNA1	HYG	no-homology *HYG* DNA donor	19	3	16	19	18	2	2	14	1	3	10	rep2-JHF1	Fig2f, FigS4a and FigS7a
O-137	*BUF1* -gRNA1	HYG	no-homology *HYG* DNA donor	19	5	14	19	15	6	1	8	1	1	6	rep3-JHF3	Fig2f, FigS4b and FigS7a
O-137	*BUF1* -gRNA1	HYG	no-homology *HYG* DNA donor	22	6	16	22	18	6	2	10	1	3	6	rep4-JHJ1	Fig2f, FigS4c and and FigS7a
O-137	*BUF1* -gRNA2	HYG	no-homology *HYG* DNA donor	9	6	3	9	3	2	0	1	0	0	1	rep1	Fig2f, FigS5a and FigS17b
O-137	*BUF1* -gRNA2	HYG	no-homology *HYG* DNA donor	22	2	20	22	20	0	2	18	3	1	14	rep2-JHEE2	Fig2f, FigS5b and FigS7b
O-137	*BUF1* -gRNA2	HYG	no-homology *HYG* DNA donor	22	1	21	22	21	0	1	20	5	7	8	rep3-JHCC5	Fig2f, FigS5c and FigS7b
O-137	*BUF1* -gRNA1&2	HYG	no-homology *HYG* DNA donor	16	4	12	16	12	7	0	5	0	1	4	rep1	Fig2d,e,f and FigS17b
O-137	*BUF1* -gRNA1&2	HYG	no-homology *HYG* DNA donor	22	12	10	22	10	1	2	7	1	0	6	rep2-JHAA7	Fig2f, FigS6a and FigS7c
O-137	*BUF1* -gRNA1&2	HYG	no-homology *HYG* DNA donor	22	0	22	22	22	3	0	19	6	3	10	rep3-JHCC6	Fig2f and FigS6b
O-137	N/A	HYG	no-homology *HYG* DNA donor	18	18	0	8	N/A	N/A	N/A	N/A	N/A	N/A	N/A	rep1	Fig2f and FigS6c
O-137	N/A	HYG	no-homology *HYG* DNA donor	6	6	0	6	N/A	N/A	N/A	N/A	N/A	N/A	N/A	rep2-JHAA8	Fig2f and FigS6d
O-137	N/A	HYG	no-homology *HYG* DNA donor	6	6	0	6	N/A	N/A	N/A	N/A	N/A	N/A	N/A	rep3-JHCC7	Fig2f and FigS6e

raw data for no-homology assay targeting *BUF1* with hygromycin selection

Isolate	gRNA used	Selection	Donor DNA	# of HYGR transformants	# of non-buff transformants	# of buff mutants	# of PCR validated transformants	# of PCR confirmed buf1 mutants	# of simple insertion buf1 mutants	# of INDEL buf1 mutants	# of PCR-negative buf1 mutants	# of large deletion buf1 mutants	# of deletion plus insertion buf1 mutants	# of large insertion buf1 mutants	Note	Figures
Guy11	*BUF1* -gRNA1	HYG	no-homology *HYG* DNA donor	20	4	16	20	16	10	0	6	0	1	5	rep1	Fig2g, FigS8a and FigS17c
Guy11	*BUF1* -gRNA1	HYG	no-homology *HYG* DNA donor	19	9	10	19	10	9	0	1	0	0	1	rep2-JHL1	Fig2g and FigS8b, d
Guy11	*BUF1* -gRNA1	HYG	no-homology *HYG* DNA donor	19	6	13	19	14	8	1	5	0	2	3	rep3-JHL2	Fig2g and FigS8c, d
Guy11	*BUF1* -gRNA2	HYG	no-homology *HYG* DNA donor	9	5	4	9	4	1	0	3	1	0	2	rep1	Fig2g, FigS8e and FigS17c
Guy11	*BUF1* -gRNA2	HYG	no-homology *HYG* DNA donor	22	2	20	22	20	0	2	18	1	1	16	rep2-JHBB6	Fig2g, FigS8f, and FigS10
Guy11	*BUF1* -gRNA1&2	HYG	no-homology *HYG* DNA donor	12	7	5	12	5	4	0	1	0	0	1	rep1	Fig2g, FigS9a and FigS17c
Guy11	*BUF1* -gRNA1&2	HYG	no-homology *HYG* DNA donor	22	1	21	22	21	1	0	20	2	2	16	rep2-JHBB7	Fig2g and FigS9b
Guy11	N/A	HYG	no-homology *HYG* DNA donor	16	16	0	6	N/A	N/A	N/A	N/A	N/A	N/A	N/A	rep1	Fig2g and FigS9c
Guy11	N/A	HYG	no-homology *HYG* DNA donor	6	6	0	6	N/A	N/A	N/A	N/A	N/A	N/A	N/A	rep2-JHBB8	Fig2g and FigS9d

raw data for no-homology assay targeting *BUF1* with G418 selection

Isolate	gRNA used	Selection	Donor DNA	# of G418R transformants	# of non-buff transformants	# of buff mutants	# of PCR validated transformants	# of PCR confirmed buf1 mutants	# of simple insertion buf1 mutants	# of INDEL buf1 mutants	# of PCR-negative buf1 mutants	Note	Figures
O-137	*BUF1* -gRNA1	G418	no-homology *G418* DNA donor	22	11	11	11	11	1	0	10	rep1-JHC1	FigS11a and g
O-137	*BUF1* -gRNA1	G418	no-homology *G418* DNA donor	14	7	7	7	7	2	0	5	rep2-JHGG1	FigS11b and g
O-137	*BUF1* -gRNA2	G418	no-homology *G418* DNA donor	22	13	9	9	9	4	1	4	rep1-JHC2	FigS11c,g and h
O-137	*BUF1* -gRNA2	G418	no-homology *G418* DNA donor	14	9	5	5	5	0	1	4	rep2-JHGG2	FigS11d, g and h
O-137	N/A	G418	no-homology *G418* DNA donor	12	12	0	12	N/A	N/A	N/A	N/A	rep1	FigS11e and g
O-137	N/A	G418	no-homology *G418* DNA donor	6	6	0	6	N/A	N/A	N/A	N/A	rep2-JHGG3	FigS11f and g

N/A=not applicable; HYGR=hygromycin resistant; G418R= G418 resistant; rep=replication

Supplementary Table 3 Statistic summary for eleven long-read *buf1* or *bas4* assemblies

Name	Rep1-*Δbuf1* # 2	Rep1-*Δ buf1#* 4	Rep1-*Δ buf1#* 5	Rep1-*Δ buf1#* 6	Rep4-*Δbuf1* #1	Rep4-*Δbuf1* #5	Rep1-*Δ buf1#* 10	Rep4-*Δ buf1#* 13	Rep1-*Δbas4* #2	Rep1-*Δbas4* #3	Rep1-*Δbas4* #11
Coverage	16.12x	117.14 x	61.66x	50.15x	56.67x	31.64x	25.21x	58.6x	19.06x	22.52x	21.44x
Total reads (Gb)	0.72 Gb	5.27 Gb	2.77 Gb	2.25 Gb	2.55 Gb	1.42 Gb	1.13 Gb	2.63 Gb	0.85 Gb	1.01 Gb	0.96 Gb
N50 (bp)	1,337,757	4,365,759	5,150,716	4,188,193	3,420,154	2,984,647	1,562,235	3,333,742	845,321	1,845,737	849,061
Max contig size (bp)	3,786,518	9,161,586	8,262,128	5,832,330	8,120,767	4,682,997	5,117,214	5,441,580	3,751,866	5,138,628	3,189,403
# scaffold	0	0	0	0	0	0	1	1	0	0	0
# contig	182	33	24	73	64	88	134	122	232	204	263
Assembly Size (Mb)	44.8 Mb	44.3 Mb	44.4 Mb	44.3 Mb	44.6 Mb	44. 7Mb	45.0 Mb	45.5Mb	45.5 Mb	45.8 Mb	45.7 Mb
Canu version	v1.9	v1.9	v1.9	v1.9	v 2.1.1	v 2.1.1	v 2.1.1	v 2.1.1	v 2.0	v 2.0	v 2.0
Genetic background	O-137	O-137	O-137	O-137	O-137	O-137	O-137	O-137	O-137	O-137	O-137
order for synteny map	(1) in Fig3a	(4) in Fig3c	(2) in Fig3b	(3) in Fig3b	(6) in Fig3d	(5) in Fig3c	(7) in Fig3d	(8) in Fig3d	(1) in Fig5a	(2) in Fig5a	(3) in Fig5b
NCBI BioSample ID	SAMN20714321	SAMN20714322	SAMN20714323	SAMN20714324	SAMN20714326	SAMN20714327	SAMN20714325	SAMN20714328	SAMN29985039	SAMN29985040	SAMN29985041

Supplementary Table 4 Summary for no-homology DNA donor assay with four loci

raw data for no-homology assay targeting *FKBP12* with hygromycin selection

Isolate	gRNA used	Selection	Donor DNA	# of HYGR transformants	# of non-FK506R transformants	# of FK506R mutants	# of PCR validated transformants	# of PCR confirmed *fkbp12* mutants	# of simple insertion *fkbp12* mutants	# of INDEL *fkbp12* mutants	# of PCR-negative *fkbp12* mutants	# of large deletion *fkbp12* mutants	# of deletion plus insertion *fkbp12* mutants	# of large insertion *fkbp12* mutants	Note	Figures
O-137	*FKBP12* -gRNA1	HYG	no-homology *HYG* DNA donor	20	15	5	5	5	1	0	4	0	1	3	rep1-JHA5	Fig4b,c and FigS19a
O-137	*FKBP12* -gRNA1	HYG	no-homology *HYG* DNA donor	22	19	3	3	3	0	0	3	0	0	3	rep2-JHB8	Fig4b,c and FigS19a
O-137	*FKBP12* -gRNA2	HYG	no-homology *HYG* DNA donor	20	15	5	5	5	4	0	1	0	0	1	rep1-JHC7	Fig4b,c and FigS19b
O-137	*FKBP12* -gRNA2	HYG	no-homology *HYG* DNA donor	22	2	20	20	20	5	1	14	0	0	14	rep2-JHCC8	Fig4b,c and FigS19c,d
O-137	N/A	HYG	no-homology *HYG* DNA donor	6	6	0	6	N/A	N/A	N/A	N/A	N/A	N/A	N/A	rep1-JHA6	Fig4b,c and FigS19e
O-137	N/A	HYG	no-homology *HYG* DNA donor	14	14	0	14	N/A	N/A	N/A	N/A	N/A	N/A	N/A	rep2-JHB9	Fig4b,c and FigS19e

raw data for no-homology assay targeting *FTR1* with hygromycin selection

Isolate	gRNA used	Selection	Donor DNA	# of HYGR transformants	# of non-FK506R transformants	# of FK506R mutants	# of PCR validated transformants	# of PCR confirmed *ftr1* mutants	# of simple insertion *ftr1* mutants	# of INDEL *ftr1* mutants	# of PCR-negative *ftr1* mutants	# of large deletion *ftr1* mutants	# of deletion plus insertion *ftr1* mutants	# of large insertion *ftr1* mutants	Note	Figures
O-137	*FTR1* -gRNA1	HYG	no-homology *HYG* DNA donor	14	N/A	N/A	14	12	8	2	2	0	0	2	rep1-JHC10	Fig4b,c and FigS20a,b
O-137	*FTR1* -gRNA1	HYG	no-homology *HYG* DNA donor	16	N/A	N/A	16	13	4	1	8	0	3	5	rep2-JHD3	Fig4b,c and FigS20c,d
O-137	*FTR1* -gRNA2	HYG	no-homology *HYG* DNA donor	15	N/A	N/A	15	4	0	0	4	0	1	3	rep1-JHE8	Fig4b,c and FigS20e
O-137	*FTR1* -gRNA2	HYG	no-homology *HYG* DNA donor	15	N/A	N/A	15	7	6	0	1	0	0	1	rep2-JHK7	Fig4b,c and FigS20f

raw data for no-homology assay targeting *BAS4* with hygromycin selection

Isolate	gRNA used	Selection	Donor DNA	# of HYGR transformants	# of non-FK506R transformants	# of FK506R mutants	# of PCR validated transformants	# of PCR confirmed *bas4* mutants	# of simple insertion *bas4* mutants	# of INDEL *bas4* mutants	# of PCR-negative *bas4* mutants	# of large deletion *bas4* mutants	# of deletion plus insertion *bas4* mutants	# of large insertion *bas4* mutants	Note	Figures
O-137	*BAS4*- gRNA1	HYG	no-homology *HYG* DNA donor	14	N/A	N/A	14	4	0	1	3	1	2	0	rep1-JHC8	Fig4b,c and FigS21a,b
O-137	*BAS4*- gRNA1	HYG	no-homology *HYG* DNA donor	16	N/A	N/A	16	5	0	0	5	0	2	3	rep2-JHD1	Fig4b,c and FigS21c
O-137	*BAS4*- gRNA2	HYG	no-homology *HYG* DNA donor	15	N/A	N/A	15	1	0	0	1	0	1	0	rep1-JHE3	Fig4b,c and FigS21d
O-137	*BAS4*- gRNA2	HYG	no-homology *HYG* DNA donor	15	N/A	N/A	15	5	0	0	5	0	1	4	rep2-JHK8	Fig4b,c and FigS21e

raw data for no-homology assay targeting *AVRPI9* with hygromycin selection

Isolate	gRNA used	Selection	Donor DNA	# of HYGR transformants	# of non-FK506R transformants	# of FK506R mutants	# of PCR validated transformants	# of PCR confirmed *avrpi9* mutants	# of simple insertion *avrpi9* mutants	# of INDEL *avrpi9* mutants	# of PCR-negative *avrpi9* mutants	# of large deletion *avrpi9* mutants	# of deletion plus insertion *avrpi9* mutants	# of large insertion *avrpi9* mutants	Note	Figures
O-137	*AVRPI9*- gRNA1	HYG	no-homology *HYG* DNA donor	14	N/A	N/A	14	2	0	1	1	0	0	1	rep1-JHC9	Fig4b,c and FigS22a,b
O-137	*AVRPI9*- gRNA1	HYG	no-homology *HYG* DNA donor	16	N/A	N/A	16	1	0	1	0	0	0	0	rep2-JHD2	Fig4b,c and FigS22c,d
O-137	*AVRPI9*- gRNA2	HYG	no-homology *HYG* DNA donor	15	N/A	N/A	15	1	0	0	1	0	0	1	rep1-JHE6	Fig4b,c and FigS22e
O-137	*AVRPI9*- gRNA2	HYG	no-homology *HYG* DNA donor	15	N/A	N/A	15	14	6	0	8	0	1	7	rep2-JHCC9	Fig4b,c and FigS22f

N/A=not applicable, HYGR= hygromycin resistant, FK506R= FK506 resistant, rep=replication

Supplementary Table 5 Summary for second site assay with *BUF1* , *FKBP12* , *FTR1* and *BAS4*

raw data for second site assay with *BUF1*

Isolate	gRNA used	Selection	Donor DNA	# of *HYGR* transformants	# of non-buff transformants	# of buff mutants	# of PCR validated transformants	# of PCR confirmed *buf1* mutants	# of simple insertion *buf1* mutants	# of INDEL *buf1* mutants	# of PCR-negative *buf1* mutants	# of large deletion *buf1* mutants	# of deletion plus insertion *buf1* mutants	# of large insertion *buf1* mutants	Note	Figures
O-137	*BUF1*- gRNA1,SS-gRNA	HYG	SS flanked *HYG* DNA donor	14	3	11	14	14	2	4	8	5	0	3	rep1-JHZ1	Fig6b,c and FigS23a,b,c
O-137	*BUF1*- gRNA1,SS-gRNA	HYG	SS flanked *HYG* DNA donor	14	6	8	14	11	2	3	6	3	0	3	rep2-JHX1	Fig6b,c and FigS23a,b,c

raw data for second site assay with *FKBP12*

Isolate	gRNA used	Selection	Donor DNA	# of HYGR transformants	# of non-FK506R transformants	# of FK506R mutants	# of PCR validated transformants	# of PCR confirmed *fkbp12* mutants	# of simple insertion *fkbp12* mutants	# of INDEL *fkbp12* mutants	# of PCR-negative *fkbp12* mutants	# of large deletion *fkbp12* mutants	# of deletion plus insertion *fkbp12* mutants	# of large insertion *fkbp12* mutants	Note	Figures
O-137	*FKBP12* -gRNA1, SS-gRNA	HYG	SS flanked *HYG* DNA donor	22	21	1	1	1	0	0	1	0	0	1	rep1-JHZ2	Fig6b,c and FigS24a,b
O-137	*FKBP12* -gRNA1, SS-gRNA	HYG	SS flanked *HYG* DNA donor	19	19	0	0	N/A	N/A	N/A	N/A	N/A	N/A	N/A	rep2-JHX2	Fig6b,c and FigS24a,b

raw data for second site assay with *BAS4*

Isolate	gRNA used	Selection	Donor DNA	# of HYGR transformants	# of non-FK506R transformants	# of FK506R mutants	# of PCR validated transformants	# of PCR confirmed *bas4* mutants	# of simple insertion *bas4* mutants	# of INDEL *bas4* mutants	# of PCR-negative *bas4* mutants	# of large deletion *bas4* mutants	# of deletion plus insertion *bas4* mutants	# of large insertion *bas4* mutants	Note	Figures
O-137	*BAS4*- gRNA1, SS-gRNA	HYG	SS flanked *HYG* DNA donor	12	N/A	N/A	12	6	0	2	4	1	2	1	rep1-JHD5	Fig6b,c and FigS25a,b,c
O-137	*BAS4*- gRNA1, SS-gRNA	HYG	SS flanked *HYG* DNA donor	12	N/A	N/A	12	2	0	0	2	2	0	0	rep2-JHD7	Fig6b,c and FigS25a,b,

raw data for second site assay with *FTR1*

Isolate	gRNA used	Selection	Donor DNA	# of HYGR transformants	# of non-FK506R transformants	# of FK506R mutants	# of PCR validated transformants	# of PCR confirmed *ftr1* mutants	# of simple insertion *ftr1* mutants	# of INDEL *ftr1* mutants	# of PCR-negative *ftr1* mutants	# of large deletion *ftr1* mutants	# of deletion plus insertion *ftr1* mutants	# of large insertion *ftr1* mutants	Note	Figures
O-137	*FTR1* -gRNA1, SS-gRNA	HYG	SS flanked *HYG* DNA donor	12	N/A	N/A	12	4	2	2	0	0	0	0	rep1-JHD6	Fig6b,c and FigS26a,c
O-137	*FTR1* -gRNA1, SS-gRNA	HYG	SS flanked *HYG* DNA donor	12	N/A	N/A	12	5	0	2	3	0	1	2	rep2-JHD8	Fig6b,c and FigS26a,b,c

raw data for second site assay with control

Isolate	gRNA used	Selection	Donor DNA	# of HYGR transformants	# of non-FK506R/non buff transformants	# of FK506R/buff mutants	# of PCR validated transformants	# of PCR confirmed mutants	# of simple insertion mutants	# of INDEL mutants	# of PCR-negative mutants	# of large deletion mutants	# of deletion plus insertion mutants	# of large insertion mutants	Note	Figures
O-137	SS-gRNA	HYG	SS flanked *HYG* DNA donor	12	12	0	12	N/A	N/A	N/A	N/A	N/A	N/A	N/A	rep1-JHZ4	FigS27a
O-137	SS-gRNA	HYG	SS flanked *HYG* DNA donor	6	6	0	6	N/A	N/A	N/A	N/A	N/A	N/A	N/A	rep2-JHEE3	FigS27b
O-137	N/A	HYG	SS flanked *HYG* DNA donor	7	7	0	7	N/A	N/A	N/A	N/A	N/A	N/A	N/A	rep1-JHZ5	FigS27c
O-137	N/A	HYG	SS flanked *HYG* DNA donor	6	6	0	6	N/A	N/A	N/A	N/A	N/A	N/A	N/A	rep2-JHEE4	FigS27d

N/A=not applicable, HYGR = hygromycin resistant; FK506R= FK506 resistant; rep=replication

Supplementary Table 6 Summary data for FK506 direct selection

Isolate	Nuclease	gRNA used	Selection	Donor DNA	# of FK506R transformants	# of PCR validated *fkbp12* mutants	# of PCR negative *fkbp12* mutants	# of PCR positive *fkbp12* mutants	# of *fkbp12* small deletion with non-MH	# of *fkbp12* small deletion mutants with 1-2 bp MH	# of *fkbp12* small deletion with ≥3 bp MH	# of *fkbp12* small and large insertion	Note	Figures
O-137	Cas12a	*FKBP12* -gRNA1-Cas12a	FK506	N/A	25	25	1	24	5	15	1	3	rep1-JHCC1	FigS28
O-137	Cas12a	*FKBP12* -gRNA1-Cas12a	FK506	N/A	26	26	0	26	7	17	0	2	rep2-JHEE5	FigS29
O-137	Cas9	*FKBP12* -gRNA1-Cas9	FK506	N/A	23	22	2	20	4	2	3	11	rep1	FigS30
O-137	Cas9	*FKBP12* -gRNA1-Cas9	FK506	N/A	27	27	2	25	9	5	2	9	rep2	FigS31
O-137	Cas12a	*FKBP12* -gRNA2-Cas12a	FK506	N/A	25	25	0	25	11	10	2	2	rep1-JHCC12	FigS34
O-137	Cas12a	*FKBP12* -gRNA2-Cas12a	FK506	N/A	25	25	2	23	9	8	1	5	rep2-JHEE6	FigS35
Δku80	Cas12a	*FKBP12* -gRNA2-Cas12a	FK506	N/A	27	27	11	16	0	1	15	0	rep1-JHHH7	FigS36
Δku80	Cas12a	*FKBP12* -gRNA2-Cas12a	FK506	N/A	16	16	5	11	0	0	11	0	rep2-JHII9	FigS37 a and c
Δku80	Cas12a	*FKBP12* -gRNA2-Cas12a	FK506	N/A	18	18	4	14	0	0	14	0	rep3-JHII10	FigS37 b and c

N/A=not applicable; FK506R= FK506 resistant; MH=microhomology; rep=replication

Supplementary Table 7 The primers used in this study

Primer name	Primers sequences (5'-3')	Purpose
LbCas12a_crRNA universal primer	CCCTAATACGACTCACTATAGGTAATTTCTACTAAGTGTAGAT	Annealing oligo for prepare DNA template used in T7 *in vitro* transcription
LbCas12a_BUF1guide1R	TTGGGGAAGTGAGCTGAAGGTGAATCTACACTTAGTAGAAATTA	Annealing oligo for prepare DNA template for *BUF1* guide1 T7 in vitro transcription
LbCas12a_BUF1guide2R	GAACTGGCCACGGGTGTTGATGGATCTACACTTAGTAGAAATTA	Annealing oligo for prepare DNA template for *BUF1* guide2 T7 in vitro transcription
LbCas12a_FKBP12guide1R	TGGCCTTCTCGCCAACTTTCATGATCTACACTTAGTAGAAATTA	Annealing oligo for prepare DNA template for *FKBP12* guide1 T7 in vitro transcription
LbCas12a_FKBP12guide2R	ACGGAAGAGTCGAACCTGAAGAAATCTACACTTAGTAGAAATTA	Annealing oligo for prepare DNA template for *FKBP12* guide2 T7 in vitro transcription
LbCas12a_FTR1guide1R	GATGGCGGTCTCGAGGGACTCGCATCTACACTTAGTAGAAATTA	Annealing oligo for prepare DNA template for *FTR1* guide1 T7 in vitro transcription
LbCas12a_FTR1guide2R	AGGAACAGCGAATACTCCTCGCCATCTACACTTAGTAGAAATTA	Annealing oligo for prepare DNA template for *FTR1* guide2 T7 in vitro transcription
LbCas12a_BAS4guide1R	TGTTGTCGGGGTAGGTTCTGACAATCTACACTTAGTAGAAATTA	Annealing oligo for prepare DNA template for *BAS4* guide1 T7 in vitro transcription
LbCas12a_BAS4guide2R	GTAGCGTGGTTGACGGCGAAGGCATCTACACTTAGTAGAAATTA	Annealing oligo for prepare DNA template for *BAS4* guide2 T7 in vitro transcription
LbCas12a_AVRPI9guide1R	AGAACTGCATGTTGGGCTGGAAGATCTACACTTAGTAGAAATTA	Annealing oligo for prepare DNA template for *AVRPI9* guide1 T7 in vitro transcription
LbCas12a_AVRPI9guide2R	CTTTTCGACTTGGCACCAGCTATATCTACACTTAGTAGAAATTA	Annealing oligo for prepare DNA template for *AVRPI9* guide2 T7 in vitro transcription
LbCas12a_HYGinsert(SS)guide1R	GTACCTTGATGCTCTATACCTGTATCTACACTTAGTAGAAATTA	Annealing oligo for prepare DNA template for Second site guide1 T7 in vitro transcription
LbCas12a_Ku80guide1R	CGGTCGTAGTGCATAGCTTGTCCATCTACACTTAGTAGAAATTA	Annealing oligo for prepare DNA template for *KU80* guide1 T7 in vitro transcription
LbCas12a_Ku80guide2R	TAGCGAATGTCTTCTGCGAACGGATCTACACTTAGTAGAAATTA	Annealing oligo for prepare DNA template for *KU80* guide2 T7 in vitro transcription
flanked *HYG* DNA donor1F	GCAAGGTTGCCCTCGTGACTGGCGCTGGTGAGAGTCGAGTATATTGAAGG	Amplifying flanked *HYG* DNA donor1
flanked *HYG* DNA donor1R	GTGAGAGATACGCGTTAGTCACATGATGCGATGTGCCCAGTCACGACGTTGTAAA	Amplifying flanked *HYG* DNA donor1
flanked *HYG* DNA donor2F	AGGACGTCACCCCTGAGGAATTTGACCGTGAGAGTCGAGTATATTGAAGG	Amplifying flanked *HYG* DNA donor2
flanked *HYG* DNA donor2R	ATCTCGAGGTGCTTGTAGGCCTCGCGGGCGACAAACCCAGTCACGACGTTGTAAA	Amplifying flanked *HYG* DNA donor2
No-homology *HYG* DNA donor F	AGAGTCGAGTATATTGAAGG	Amplifying no-homology *HYG* DNA donor
No-homology *HYG* DNA donor R	CCCAGTCACGACGTTGTAAA	Amplifying no-homology *HYG* DNA donor
No-homology *G418* DNA donor F	TCTAGATTAACGCTTACA	Amplifying no-homology *G418* DNA donor
No-homology *G418* DNA donor R	GTCGACTCCACCGAGCAGGGTATTG	Amplifying no-homology *G418* DNA donor
BUF1 _F	GCATCCATTCCCATTCATCC	*BUF1* locus genotyping
BUF1 _R	GGGCAGCAACTGTCTCCATT	*BUF1* locus genotyping
BUF1 _upstreamF	GTTGGCACGGCACCTCTGTA	*BUF1* 5' upstream genotyping
BUF1 _upstreamR	CTGGGATCGCGTCATACTTG	*BUF1* 5' upstream genotyping
BUF1 _downstreamF	GACAGTTGCTGCCCAAAGAT	*BUF1* 3' downstream genotyping
BUF1 _downstreamR	AATGAGAAGGAGCGAGGATG	*BUF1* 3' downstream genotyping
BUF1_assembly_confirmF	GCTCTTTGTTATGCTGTCCCTG	large deletion mutation confirmation
BUF1_assembly_confirmR	TGTGCTGCTATCGTGTCCTGT	large deletion mutation confirmation
FKBP12 _F	GCAGTAGCTGGTGCTGTTTG	*FKBP12* locus genotyping
FKBP12 _R	CCTCTATACCTATGTCCCTTGTC	*FKBP12* locus genotyping
FKBP12 _upstreamF	TGTGGCTTCATTGTTGGTTT	*FKBP12* 5' upstream genotyping
FKBP12 _upstreamR	CGATGTCTGTATTTACGGGACT	*FKBP12* 5' upstream genotyping
FKBP12 _downstreamF	CTCACCAGCGATGTCTACCT	*FKBP12* 3' downstream genotyping
FKBP12 _downstreamR	AAGGGTCAAGACTATACAGAAGTA	*FKBP12* 3' downstream genotyping
FTR1 _F	ATCCGCAACTTGACAATGGA	*FTR1* locus genotyping
FTR1 _R	GGTGTAGACCGCTGGTGGTAG	*FTR1* locus genotyping
FTR1 _upstreamF	CTACCACCCATCCTCACCAA	*FTR1* 5' upstream genotyping
FTR1 _upstreamR	TCCCGACTAGAGTTCAATCTGC	*FTR1* 5' upstream genotyping
FTR1 _downstreamF	ATCCAGGCATTGGCTTGATAC	*FTR1* 3' downstream genotyping
FTR1 _downstreamR	GCACGCTGTAATGATTTGTCG	*FTR1* 3' downstream genotyping
BAS4 _F	AAACCTCGGTATTCTATGCG	*BAS4* locus genotyping
BAS4 _R	TCCCTAATTCCCAGTCCAGT	*BAS4* locus genotyping
BAS4 _upstreamF	GCCACAGGGCAACTTAGCAT	*BAS4* 5' upstream genotyping
BAS4 _upstreamR	TTTCATCTCGTCCGCATAGA	*BAS4* 5' upstream genotyping
BAS4 _downstreamF	TGGACTGGGAATTAGGGAGA	*BAS4* 3' downstream genotyping
BAS4 _downstreamR	AATGGTTCATGCGGAGTTGG	*BAS4* 3' downstream genotyping
*AVRPI9*_ F	ACTTCCTCTGCTCCTTCCTA	*AVRPI9* locus genotyping
AVRPI9 _R	GCTTGCAGAAGAGTTATTTATT	*AVRPI9* locus genotyping
*AVRPI9*_ upstreamF	CCTTTCCAGAATCGCTACAT	*AVRPI9* 5' upstream genotyping
AVRPI9 _upstreamR	GTCGAACAGGTGCCCAATAT	*AVRPI9* 5' upstream genotyping
AVRPI9 _downstreamF	CTTACCATCGCCGCAACCAT	*AVRPI9* 3' downstream genotyping
AVRPI9 _downstreamR	CGTGGCACTTTGGACACTCG	*AVRPI9* 3' downstream genotyping
HYGinsert(SS)_F	TGTCTGTGGGTTACCTATGC	Second site locus genotyping
HYGinsert(SS)_R	ATCACCGTCGTTCACTTTCT	Second site locus genotyping
HYGinsert(SS)KpnI_F	CGGGGTACCGGGTAAAGAGGCTTGTCCAT	Cloning Ss flanked *HYG* DNA donor
HYGinsert(SS)XbaI_R	GCTCTAGACAGAGCGAACACGAGATTGT	Cloning Ss flanked *HYG* DNA donor
HYGinsert(SS)SalI_F	ACGCGTCGACAGATGCGTAACAGGACTCGG	Cloning Ss flanked *HYG* DNA donor
HYGinsert(SS)PstI_R	AACTGCAGGCTTTCCAGCTCCCATGACC	Cloning Ss flanked *HYG* DNA donor
BUF1 _junctionSF	ATGCCTGCCGTCACTCAACC	Testing the sequenes in *BUF1* intergration junction
BUF1 _junctionSR	GCGCTTCCGAGTTACGTGTT	Testing the sequenes in *BUF1* intergration junction
MOACTINF	CCGTGACTTGACCGACTACCTGA	Loading control
MOACTINR	AGAGCGAGGCGAGAATGGAAC	Loading control
KU80 _KO_up_F	CAGCGATGATATGCCGTGAA	Constructing long-homology *G418* donor for *KU80* deletion
KU80 _KO_up_R	TGGAAATTGTAAGCGTTAATCTAGAAACAGGCAGATCAACCACCC	Constructing long-homology *G418* donor for *KU80* deletion
KU80 _KO_down_F	TTCTTGACGAGTTCTTCTGAGGCTGGGATTATTGTTTGGC	Constructing long-homology *G418* donor for *KU80* deletion
KU80 _KO_down_R	GGCGTCTGAAAGTACGGAGA	Constructing long-homology *G418* donor for *KU80* deletion
G418 _Front_F1	TCTAGATTAACGCTTACAATTTCCA	Constructing long-homology *G418* donor for *KU80* deletion
G418 _Front_R1	GCCCAATAGCAGCCAGTCC	Constructing long-homology *G418* donor for *KU80* deletion
G418 _Back_F2	CAACAACACGCATCATCCCA	Constructing long-homology *G418* donor for *KU80* deletion
G418 _Back_R2	TCAGAAGAACTCGTCAAGAA	Constructing long-homology *G418* donor for *KU80* deletion
KU80 _inside_F	CTTGAAGACGGCGAGGAATT	Genotyping *KU80* deletion mutants
KU80 _inside_R	CCAGTGGCGGAAACTGATAG	Genotyping *KU80* deletion mutants
KU80 _outside_1F	TGTTGATTTGGATCTGGCTAA	Genotyping *KU80* deletion mutants
KU80 _outside_1R	ATACCGTAAAGCACGAGGAA	Genotyping *KU80* deletion mutants
KU80 _outside_2F	ATGAACTGCAAGACGAGGCA	Genotyping *KU80* deletion mutants
KU80 _outside_2R	GCGTTGTGAAGATACCGATGAC	Genotyping *KU80* deletion mutants
FKBP12 _KO_up_F	TGACATTGCTTGCACGAGAT	Constructing long-homology *HYG* donor for *FKBP12* deletion
FKBP12 _KO_up_R	CTCGACTCTAGAACTAGTGGATCCATAAGTGGCTTTGAAGAACG	Constructing long-homology *HYG* donor for *FKBP12* deletion
FKBP12 _KO_down_F	GGAACCAGTCGACCTGCAGATCCGATGACCTTATGCTTTG	Constructing long-homology *HYG* donor for *FKBP12* deletion
FKBP12 _KO_down_R	CAGTCGTCCTGTCGTGGTTC	Constructing long-homology *HYG* donor for *FKBP12* deletion
HYG _KO_FRONT_F2	GGATCCACTAGTTCTAGAGTCGAG	Constructing long-homology *HYG* donor for *FKBP12* deletion
HYG _KO_FRONT_R	CGATTCCTTGCGGTCCGAA	Constructing long-homology *HYG* donor for *FKBP12* deletion
HYG _KO_BACK_F	GATGTAGGAGGGCGTGGATATGTC	Constructing long-homology *HYG* donor for *FKBP12* deletion
HYG _KO_BACK_R2	TCTGCAGGTCGACTGGTTCC	Constructing long-homology *HYG* donor for *FKBP12* deletion
FKBP12 _ins_detect_F1	GGGTGTTACAAAGACCACAA	Sanger sequencing *fkbp12* mutants
