# Supplementary material for: CRISPR-Cas12a induced DNA double-strand breaks are repaired by multiple pathways with different mutation profiles in Magnaporthe oryzae
Source: Nat Commun. 2022 Nov 22;13:7168. doi: 10.1038/s41467-022-34736-1 (PMC9684475; doi:10.1038/s41467-022-34736-1)
Supplement: Supplementary file 3 — Reporting Summary [file 41467_2022_34736_MOESM3_ESM.pdf]

nature portfolio

Corresponding author(s): David E. Cook

Last updated by author(s): Oct 26, 2022

Reporting Summary

Nature Portfolio wishes to improve the reproducibility of the work that we publish. This form provides structure for consistency and transparency in reporting. For further information on Nature Portfolio policies, see our Editorial Policies and the Editorial Policy Checklist.

Statistics

For all statistical analyses, confirm that the following items are present in the figure legend, table legend, main text, or Methods section.

n/a	Confirmed	
☐	☒	The exact sample size (*n*) for each experimental group/condition, given as a discrete number and unit of measurement
☐	☒	A statement on whether measurements were taken from distinct samples or whether the same sample was measured repeatedly
☐	☒	The statistical test(s) used AND whether they are one- or two-sided<br>*Only common tests should be described solely by name; describe more complex techniques in the Methods section.*
☒	☐	A description of all covariates tested
☒	☐	A description of any assumptions or corrections, such as tests of normality and adjustment for multiple comparisons
☐	☒	A full description of the statistical parameters including central tendency (e.g. means) or other basic estimates (e.g. regression coefficient) AND variation (e.g. standard deviation) or associated estimates of uncertainty (e.g. confidence intervals)
☐	☒	For null hypothesis testing, the test statistic (e.g. *F*, *t*, *r*) with confidence intervals, effect sizes, degrees of freedom and *P* value noted<br>*Give P values as exact values whenever suitable.*
☒	☐	For Bayesian analysis, information on the choice of priors and Markov chain Monte Carlo settings
☒	☐	For hierarchical and complex designs, identification of the appropriate level for tests and full reporting of outcomes
☒	☐	Estimates of effect sizes (e.g. Cohen's *d*, Pearson's *r*), indicating how they were calculated

Our web collection on statistics for biologists contains articles on many of the points above.

Software and code

Policy information about availability of computer code

Data collection	Nanopore MinION platform was used for collecting the long-read sequencing data.<br>Please see more detailed information for commercial used in this study in the Materials and Methods section.
Data analysis	Guppy (version 3.4.4)<br>Porechop (version 0.2.4)<br>Canu (version 1.9 , 2.0 and 2.2.1)<br>Integrative Genomics Viewer (IGV) (version 2.5.0)<br>samtools (version 1.9)<br>minimap2 (version 2.17-r941)<br>Easyfig (version 2.2.5)<br>RStudio (Version 1.2.5001)<br>TBtools (Version 1.068)<br>MEGA X (version version 10.0.1)<br>GraphPad Prism (Version 8.0.1 or 9)<br>Adobe Illustrator (Version 25.4.1)<br>were used for data analysis.<br>Please see more detailed information for the software used in this study in the Materials and Methods section.

For manuscripts utilizing custom algorithms or software that are central to the research but not yet described in published literature, software must be made available to editors and reviewers. We strongly encourage code deposition in a community repository (e.g. GitHub). See the Nature Portfolio guidelines for submitting code & software for further information.

Data

Policy information about availability of data

All manuscripts must include a data availability statement. This statement should provide the following information, where applicable:

- Accession codes, unique identifiers, or web links for publicly available datasets
- A description of any restrictions on data availability
- For clinical datasets or third party data, please ensure that the statement adheres to our policy

The de novo assemblies of Cas12a-edited strains from nanopore sequencing have been deposited in the WGS Genome of the National Center for Biotechnology Information (NCBI) under the BioProject accession No. PRJNA753862. The base called nanopore reads after adapter removal have also been deposited under the same BioProject accession No. PRJNA753862 with the Short Read Archive (SRA) accession No. SRR15459267 to SRR15459274 and No. SRR20662591 to SRR20662593. Source data are provided with this paper.
Raw gel images are provided in a supplementary file.
Raw data for Fig1f,g, Fig2f,g, Fig4b,c, Fig6b,c, Fig7b,d, e, Fig8a,b,d,e, FigS11g and FigS17d are provided in the Source Data file.
Gene sequence with MGG_number is from 70-15 MG8 genome annotation and can be found in FungiDB (https://fungidb.org/fungidb/app). The sequence of polq homologs can be found in NCBI Genbank (https://www.ncbi.nlm.nih.gov/genbank/).
M. oryzae field isolates O-137, Guy11 and derived mutants within this study are available upon reasonable request and may require permit.

Field-specific reporting

Please select the one below that is the best fit for your research. If you are not sure, read the appropriate sections before making your selection.

☒ Life sciences ☐ Behavioural & social sciences ☐ Ecological, evolutionary & environmental sciences

For a reference copy of the document with all sections, see nature.com/documents/nr-reporting-summary-flat.pdf

Life sciences study design

All studies must disclose on these points even when the disclosure is negative.

Sample size	No sample size calculation was performed. All the experiments were performed two to four times independently to determine signal from noise and assess variation, which is widely accepted in our field (Foster et al., 2018, Scientific reports; Wang et al., 2018, Fungal Genetics and Biology and Ah-Fong et al., 2021, Molecular Plant Pathology). Exact sample size and number of replication were indicated in the manuscript.
Data exclusions	No data were excluded.
Replication	The fungal transformations were repeated between 2 and 4 times depending on the experiment. This information is included in the figure legend for each figure. The transformations were repeated on independent days using independent material. Results across all the individuals collected from independent experiments were combined for analysis.<br>Please see more details in the Supplementary Table S1,S2,S4,S5 and S6.
Randomization	Colonies that displayed the simple insertion genotype were randomly selected for Sanger sequencing in Fig2h. For long read sequencing and assembly shown in Figure 3 and 5, individuals transformants were randomly sampled from the PCR genotyping results. The ku80 deletion mutant (Δku80#9single spore#8) was randomly selected from a plate of transformants for molecular characterization in Fig8.<br>All above described randomizations occurred within one single group and did not involve further group comparison. PCR genotyping analysis used all recovered transformants and did not require randomization.
Blinding	The experiments did not use blinding during their design or execution.

Reporting for specific materials, systems and methods

We require information from authors about some types of materials, experimental systems and methods used in many studies. Here, indicate whether each material, system or method listed is relevant to your study. If you are not sure if a list item applies to your research, read the appropriate section before selecting a response.

Materials & experimental systems

n/a	Involved in the study
☒	☐ Antibodies
☒	☐ Eukaryotic cell lines
☒	☐ Palaeontology and archaeology
☐	☒ Animals and other organisms
☒	☐ Human research participants
☒	☐ Clinical data
☒	☐ Dual use research of concern

Methods

n/a	Involved in the study
☒	☐ ChIP-seq
☒	☐ Flow cytometry
☒	☐ MRI-based neuroimaging

Animals and other organisms

Policy information about studies involving animals; ARRIVE guidelines recommended for reporting animal research

Laboratory animals	This study did not involve any laboratory animals.<br>Only Magnaporthe oryzae (plant pathogenic fungus) is involved.
Wild animals	*Provide details on animals observed in or captured in the field; report species, sex and age where possible. Describe how animals were caught and transported and what happened to captive animals after the study (if killed, explain why and describe method; if released, say where and when) OR state that the study did not involve wild animals.*
Field-collected samples	Filter papers with wild-type Magnaporthe oryzae are stored in -20c freezer.
Ethics oversight	*Identify the organization(s) that approved or provided guidance on the study protocol, OR state that no ethical approval or guidance was required and explain why not.*

Note that full information on the approval of the study protocol must also be provided in the manuscript.

March 2021
